# Supplementary material for: Implementation of an electronic patient-reported measure of barriers to antiretroviral therapy adherence with the Opal patient portal: Protocol for a mixed method type 3 hybrid pilot study at a large Montreal HIV clinic
Source: PLoS One. 2021 Dec 30;16(12):e0261006. doi: 10.1371/journal.pone.0261006 (PMC8717992; doi:10.1371/journal.pone.0261006)
Supplement: S1 Checklist — (DOC) [file pone.0261006.s001.doc]

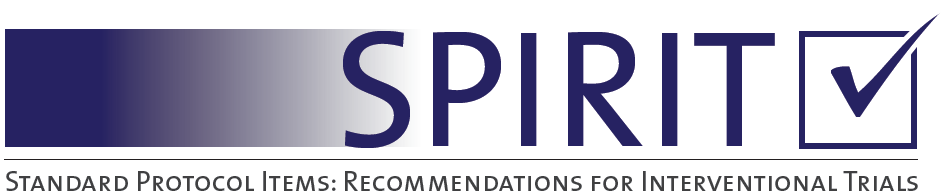


SPIRIT 2013 Checklist: Recommended items to address in a clinical trial protocol and related documents*

| Section/item | | Status | ItemNo | Description |
| --- | --- | --- | --- | --- |
| **Administrative information** | | | | |
| Title | | √ | 1 | Descriptive title identifying the study design, population, interventions, and, if applicable, trial acronym  **Response**: See title |
| Trial registration | | √ | 2a | Trial identifier and registry name. If not yet registered, name of intended registry  **Response**: The trial registry is ClinicalTrials.gov and the identifier is NCT04702412. |
|  |  |  | 2b | All items from the World Health Organization Trial Registration Data Set  **Response**: A table with responses to these items appears in the Supplementary material. |
| Protocol version | | √ | 3 | Date and version identifier  **Response**: The manuscript is consistent with the REB approved protocol, version Dec 16, 2020. |
| Funding | | √ | 4 | Sources and types of financial, material, and other support  **Response**: See especially section entitled ‘Current status’. |
| Roles and responsibilities | | √ | 5a | Names, affiliations, and roles of protocol contributors  **Response**: See the title page and the section ‘Author contributions’. |
|  |  | √ | 5b | Name and contact information for the trial sponsor  **Response**: the sponsor is the Research Institute of the McGill University Health Centre (RI-MUHC), 2155, Guy street, 5th floor, Montreal, QC, H3H 2R9 |
|  | |  | 5c | Role of study sponsor and funders, if any, in study design; collection, management, analysis, and interpretation of data; writing of the report; and the decision to submit the report for publication, including whether they will have ultimate authority over any of these activities  **Response**: The sponsor (RI-MUHC) and the funder, Merck Canada Inc., had and will have no role in any of these aspects. The funder, CTN, will provide assistance with data collection by creating the CRFs, for instance, and providing feedback on the statistical analysis plan. For full details, see response below. The CTN will have no decisional authority on the submission of reports for publication or conference presentation. |
|  | |  | 5d | Composition, roles, and responsibilities of the coordinating centre, steering committee, endpoint adjudication committee, data management team, and other individuals or groups overseeing the trial, if applicable (see Item 21a for data monitoring committee)  **Response**: The CIHR HIV Clinical Trials Network (CTN), which is funding the study, will provide assistance with Case Report Form Development, Database Set-up, Data Validation Plan Preparation, and Data and Safety Monitoring Committee Management. For details on the steering committee see trial protocol. |
| Introduction | |  |  |  |
| Background and rationale | | √ | 6a | Description of research question and justification for undertaking the trial, including summary of relevant studies (published and unpublished) examining benefits and harms for each intervention.  **Response**: See especially the introduction. |
|  |  | n.a. | 6b | Explanation for choice of comparators |
| Objectives | | √ | 7 | Specific objectives or hypotheses  **Response**: See the section ‘Aims and objectives’ |
| Trial design | | √ | 8 | Description of trial design including type of trial (eg, parallel group, crossover, factorial, single group), allocation ratio, and framework (eg, superiority, equivalence, noninferiority, exploratory)  **Response**: See the section ‘Study design’. |
|  | Methods: Participants, interventions, and outcomes | | | |
| Study setting | | √ | 9 | Description of study settings (eg, community clinic, academic hospital) and list of countries where data will be collected. Reference to where list of study sites can be obtained.  **Response**: See the section ‘Setting and participants’. |
| Eligibility criteria | | √ | 10 | Inclusion and exclusion criteria for participants. If applicable, eligibility criteria for study centres and individuals who will perform the interventions (eg, surgeons, psychotherapists).  **Response**: See the section ‘Setting and participants’. |
| Interventions | | √ | 11a | Interventions for each group with sufficient detail to allow replication, including how and when they will be administered  **Response**: See especially the section ‘The I-Score intervention’. |
|  |  | √ | 11b | Criteria for discontinuing or modifying allocated interventions for a given trial participant (eg, drug dose change in response to harms, participant request, or improving/worsening disease)  **Response**: See the exclusion criteria in the section ‘Settings and participants’. |
|  |  | √ | 11c | Strategies to improve adherence to intervention protocols, and any procedures for monitoring adherence (eg, drug tablet return, laboratory tests)  **Response**: See the section entitled ‘The implementation strategy’. |
|  |  | n.a. | 11d | Relevant concomitant care and interventions that are permitted or prohibited during the trial |
| Outcomes | | √ | 12 | Primary, secondary, and other outcomes, including the specific measurement variable (eg, systolic blood pressure), analysis metric (eg, change from baseline, final value, time to event), method of aggregation (eg, median, proportion), and time point for each outcome. Explanation of the clinical relevance of chosen efficacy and harm outcomes is strongly recommended  **Response**: See especially Table 2. |
| Participant timeline | | √ | 13 | Time schedule of enrolment, interventions (including any run-ins and washouts), assessments, and visits for participants. A schematic diagram is highly recommended (see Figure)  **Response**: See especially Fig 3. |
| Sample size | | √ | 14 | Estimated number of participants needed to achieve study objectives and how it was determined, including clinical and statistical assumptions supporting any sample size calculations.  **Response**: See the section ‘Setting and participants’ for the sample size justification. |
| Recruitment | | √ | 15 | Strategies for achieving adequate participant enrolment to reach target sample size  **Response**: See especially the section ‘Recruitment and consent process’. |
|  | **Methods: Assignment of interventions (for controlled trials)** | | | |
| Allocation: | |  |  |  |
| Sequence generation | | n.a. | 16a | Method of generating the allocation sequence (eg, computer-generated random numbers), and list of any factors for stratification. To reduce predictability of a random sequence, details of any planned restriction (eg, blocking) should be provided in a separate document that is unavailable to those who enrol participants or assign interventions |
| Allocation concealment mechanism | | n.a. | 16b | Mechanism of implementing the allocation sequence (eg, central telephone; sequentially numbered, opaque, sealed envelopes), describing any steps to conceal the sequence until interventions are assigned |
| Implementation | | n.a. | 16c | Who will generate the allocation sequence, who will enrol participants, and who will assign participants to interventions |
| Blinding (masking) | | n.a. | 17a | Who will be blinded after assignment to interventions (eg, trial participants, care providers, outcome assessors, data analysts), and how |
|  | | n.a. | 17b | If blinded, circumstances under which unblinding is permissible, and procedure for revealing a participant’s allocated intervention during the trial |
|  | **Methods: Data collection, management, and analysis** | | | |
| Data collection methods | | √ | 18a | Plans for assessment and collection of outcome, baseline, and other trial data, including any related processes to promote data quality (eg, duplicate measurements, training of assessors) and a description of study instruments (eg, questionnaires, laboratory tests) along with their reliability and validity, if known. Reference to where data collection forms can be found, if not in the protocol.  **Response**: See especially the section entitled ‘Data collection’. See also the Appendices for the study questionnaires. |
|  | | √ | 18b | Plans to promote participant retention and complete follow-up, including list of any outcome data to be collected for participants who discontinue or deviate from intervention protocols.  **Response**: See especially Table 2. |
| Data management | |  | 19 | Plans for data entry, coding, security, and storage, including any related processes to promote data quality (eg, double data entry; range checks for data values). Reference to where details of data management procedures can be found, if not in the protocol.  **Response**: See response to 5d. Several methods will be applied by the statistician (SV) to promote data quality. For example, he will visually inspect the data, calculate the range for each variable, ensure that the maximum and minimum scores are coherent, create boxplots to detect outliers, examine if there is duplicate data. See also the ‘Data management’ and the ‘Opal cybersecurity’ sections. |
| Statistical methods | | √ | 20a | Statistical methods for analysing primary and secondary outcomes. Reference to where other details of the statistical analysis plan can be found, if not in the protocol  **Response**: See section entitled “Data analysis”. |
|  | | √ | 20b | Methods for any additional analyses (eg, subgroup and adjusted analyses)  **Response**: In the above section, details are also provided on the qualitative analyses. |
|  | | √ | 20c | Definition of analysis population relating to protocol non-adherence (eg, as randomised analysis), and any statistical methods to handle missing data (eg, multiple imputation)  **Response**: All analyses based on regression models will be made with mixed models, due to their advantages for handling missing data. All subjects will be included in the analyses and data values corresponding to missing time points (T1, T2 or T3) will be omitted. Concerning the analyses based on hypotheses testing, such as Student’s t-tests and McNemar tests, missing data will be omitted. Finally, all missing data will be included and summarized in the descriptive statistics. |
|  | **Methods: Monitoring** | | | |
| Data monitoring | |  | 21a | Composition of data monitoring committee (DMC); summary of its role and reporting structure; statement of whether it is independent from the sponsor and competing interests; and reference to where further details about its charter can be found, if not in the protocol. Alternatively, an explanation of why a DMC is not needed.  **Response**: Data and Safety Monitoring Committee Management will be undertaken by the CTN (study funders) which is independent from the sponsor and has no competing interests. |
|  | | n.a. | 21b | Description of any interim analyses and stopping guidelines, including who will have access to these interim results and make the final decision to terminate the trial |
| Harms | | √ | 22 | Plans for collecting, assessing, reporting, and managing solicited and spontaneously reported adverse events and other unintended effects of trial interventions or trial conduct  **Response:** Participants in this study, whether they are involved in the interview or in filling out the study questionnaires, face no direct physical risk of participating as they will not be submitted to any pharmaceutical or invasive medical interventions. If an adverse event (AE) occurs, it is probable that it will involve a negative emotional reaction (e.g., distress, anxiety) caused by the qualitative interview about the intervention or by completing the I-Score PROM (e.g., on difficulties adhering to ART). Participants will be informed that they can be referred to a mental health professional within a reasonable amount of time should they experience significant emotional distress due to their participation.  Any AE that occurs from the moment a participant signs the consent form to the time they leave the study will be documented by the study’s research personnel in the appropriate Case Report Form. The dates of the beginning and end of the AE, its severity (Grade), and the principal investigator’s judgment about the relationship of the AE to participation in the study (e.g., “definitely related” to “not related”) will be recorded on this form. Serious Adverse Events will be reported as per Standard Operating Procedures at the MUHC. |
| Auditing | | √ | 23 | Frequency and procedures for auditing trial conduct, if any, and whether the process will be independent from investigators and the sponsor  **Response**: Since this is not a Health Canada regulated study, the CTN will not be auditing this trial. The Quality Assurance Department of the Research Institute of the McGill University Health Centre could possibly audit this study and the process would be independent from the investigators and sponsor. |
|  | Ethics and dissemination | | | |
| Research ethics approval | | √ | 24 | Plans for seeking research ethics committee/institutional review board (REC/IRB) approval  **Response**: Approval of the study (CTNPT039 / 2021-  7190) was granted by the McGill University Health Centre Research Ethics Board January 18, 2021. |
| Protocol amendments | |  | 25 | Plans for communicating important protocol modifications (eg, changes to eligibility criteria, outcomes, analyses) to relevant parties (eg, investigators, REC/IRBs, trial participants, trial registries, journals, regulators)  **Response:** Protocol amendments will be submitted to the REB of the McGill University Health Centre for approval through the MUHC Nagano platform. The Project Manager of the Sponsor (CTN) will also be informed as will all study participants, collaborators, and investigators affected by the amendment. The clinicaltrials.gov record of the study will also be updated. If changes are made to the protocol that affects the participants or changes are made to the consent form, enrolled patients will have to reconsent to the study. |
| Consent or assent | | √ | 26a | Who will obtain informed consent or assent from potential trial participants or authorised surrogates, and how (see Item 32)  **Response**: See section entitled ‘Recruitment and consent process’. See also the consent form in the Appendices. |
|  | | n.a. | 26b | Additional consent provisions for collection and use of participant data and biological specimens in ancillary studies, if applicable |
| Confidentiality | |  | 27 | How personal information about potential and enrolled participants will be collected, shared, and maintained in order to protect confidentiality before, during, and after the trial  **Response**: All of the study’s paper documents containing nominal information on the participants will be stored under lock and key (e.g., consent forms) and they will only be accessible to the project investigators and personnel. The questionnaires and audio recordings of the interviews and focus groups will be identified by an alphanumeric code, assigned to each participant. All electronic files (e.g., interview transcriptions) will also be identified by a code and will require a password to access them. All electronic study data will be stored on a local server protected by the MUHC. The computers used for analysis or record-keeping will also be password protected.  When the study is over, all study documents and data will be kept in a secure place at the Chronic Viral Illness Service of the McGill University Health Centre for 2 years. Afterwards, they will be archived at Iron Mountain in Laval, Quebec. Once the study’s results have been published, they will be kept there for 7 years.  See also the ‘Data management’ section, the consent form in the Appendices, and the section entitled ‘Opal cybersecurity’. |
| Declaration of interests | | √ | 28 | Financial and other competing interests for principal investigators for the overall trial and each study site  **Response**: The PI has no competing interests. |
| Access to data | | √ | 29 | Statement of who will have access to the final trial dataset, and disclosure of contractual agreements that limit such access for investigators  **Response**: No contractual agreements limit access to CTNPT039 data for investigators. The final quantitative trial dataset will be made available to interested parties. |
| Ancillary and post-trial care | | n.a. | 30 | Provisions, if any, for ancillary and post-trial care, and for compensation to those who suffer harm from trial participation |
| Dissemination policy | | √ | 31a | Plans for investigators and sponsor to communicate trial results to participants, healthcare professionals, the public, and other relevant groups (eg, via publication, reporting in results databases, or other data sharing arrangements), including any publication restrictions  **Response**: The results of this study will be published in peer-reviewed journals and presented at Academic Rounds of the McGill University Health Centre and conferences. The clinicaltrials.gov record of our study will be updated with all publications. The sponsor (CTN) will report study publications in their newsletter. Publications will also be signalled to the Department of Family Medicine at McGill University and reported in their newsletter. |
|  | | √ | 31b | Authorship eligibility guidelines and any intended use of professional writers  **Response**: There will be no use of professional writers. Authorship eligibility will follow the criteria of the International Committee of Medical Journal Editors. |
|  | |  | 31c | Plans, if any, for granting public access to the full protocol, participant-level dataset, and statistical code  **Response**: The quantitative dataset and well as the statistical code will be made available to interested parties at study end. |
| Appendices | |  |  |  |
| Informed consent materials | |  | 32 | Model consent form and other related documentation given to participants and authorised surrogates  **Response**: One of the study’s consent forms is provided as supplementary material as are two of the study questionnaires. |
| Biological specimens | | n.a. | 33 | Plans for collection, laboratory evaluation, and storage of biological specimens for genetic or molecular analysis in the current trial and for future use in ancillary studies, if applicable |

*It is strongly recommended that this checklist be read in conjunction with the SPIRIT 2013 Explanation & Elaboration for important clarification on the items. Amendments to the protocol should be tracked and dated. The SPIRIT checklist is copyrighted by the SPIRIT Group under the Creative Commons “[Attribution-NonCommercial-NoDerivs 3.0 Unported](http://www.creativecommons.org/licenses/by-nc-nd/3.0/)” license.
