# Supplementary material for: Implementation of an electronic patient-reported measure of barriers to antiretroviral therapy adherence with the Opal patient portal: Protocol for a mixed method type 3 hybrid pilot study at a large Montreal HIV clinic
Source: PLoS One. 2021 Dec 30;16(12):e0261006. doi: 10.1371/journal.pone.0261006 (PMC8717992; doi:10.1371/journal.pone.0261006)
Supplement: S1 Table — PROM = patient-reported outcome measure; CFIR = Consolidated Framework for Implementation Research; a Reproduced or adapted from Foster et al. [41]; b Based on Damschroder et al. [25]; c Based on the taxonomies of Powell et al. [42,43]. (DOCX) [file pone.0261006.s002.docx]

| **Stage of PROM implementa-tion** ^a^ | **Facilitator/ barrier** ^a^ | **CFIR framework construct** ^b^ | **Addressed** | |
| --- | --- | --- | --- | --- |
|  |  |  | Prior to the pilot | Within the pilot:  Implementation strategy ^c^ |
| **Purpose**: Motivations for implementing and objectives of using PROM | The perceived reason for implementing a PROM is important -if external, impact can be mixed | Intervention characteristics: Intervention source  Outer setting:  External policy (e.g. guidelines) | Both individuals with HIV and providers in Canada and France were involved throughout the development of the PROM, including in needs assessments and content validation. | Educate: Conduct educational meetings  *An educational meeting will cover the origins of the PROM and how it was shaped through stakeholder engagement. The PROM intervention will also be presented as consistent with HIV treatment guidelines on the monitoring of adherence barriers.* |
|  | The PROM should be to support the management of patients, rather than solely used as an audit tool | Inner setting: Implementation climate-Compatibility | The measure’s main purpose is to aid in patient management and secondarily, it will serve as a research tool. It is not designed for use in performance management. | Educate: Conduct educational meetings  *The educational meeting will emphasize the patient management function of the PROM, clearly establishing that results are not used to monitor clinician performance, despite the collection of a service outcome in the pilot.* |
| **Designing**: Deciding the PROM process and how to implement it | Involve clinicians and patients in designing the PROM process | Intervention characteristics: Intervention source  Intervention characteristics: Design quality | Both individuals with HIV and providers in Canada and France were involved throughout the development of the PROM, including in needs assessments and content validation.  This pilot study is conducted in part so that the PROM process can be adapted to local patient and physician concerns. | Plan: Build buy-in -Involve patients/ consumers  Plan: Organize clinician implementation team meetings  *Participants will have several opportunities to provide feedback on the intervention and its implementation. Physicians will participate in focus groups (T2, T3) and patients will participate in individual interviews (T2, T3).* |
|  | Consider the needs and resources of clinicians and patients when designing the PROM process | External setting: Patient needs and resources  Inner setting: Implementation climate-Compatibility | Both individuals with HIV and providers in Canada and France were involved throughout the development of the PROM, including in needs assessments and in content validation with Delphi techniques. Patients and providers were also involved in designing the smartphone application to administer the PROM. | Plan: Build buy-in -Involve patients/ consumers  Plan: Organize clinician implementation team meetings  Educate: Conduct ongoing training  Educate: Provide ongoing consultation  *These educational strategies will be enacted through the hiring of a* *Application Manager who will train and assist patients and providers in the PROM process. Patients will be individually trained.* |
|  | The PROM process should limit complexity and have compatibility with current ways of working and clinicians’ values | Intervention characteristics: Complexity  Inner setting: Implementation climate-Compatibility | See above response.  In addition, PROM completion and results examination were scheduled to coincide with regular clinic appointment times, to limit disruption.  The use of a smartphone app was also chosen to simplify the PROM process. | Plan: Build buy-in -Involve patients/ consumers  Plan: Organize clinician implementation team meetings  Educate: Conduct ongoing training  Educate: Provide ongoing consultation |
|  | Have adaptability within the PROM process | Intervention characteristics: Adaptability | - | Promote adaptability  *We have distinguished between the core components of the intervention and those aspects which can be tailored to local needs.*  Quality management: Conduct cyclical small tests of change  *Building on feedback received through participating patient and physician feedback and the Application Manager’s field notes, we will use a rapid-cycle evaluation approach, using the CFIR, to improve implementation.* |
|  | Choose PROMs which are perceived as relevant and appropriate | Intervention characteristics: Evidence strength and quality  Characteristics of individuals: Knowledge and beliefs about the intervention | The Delphi which consulted individuals with HIV and providers on the actionability, and relevance of the PROM’s content served in the final selection of the PROM’s items. | Educate: Conduct educational meetings  *The educational meeting will provide data on the perceived importance and relevance of the PROM, including the results of the Delphi.* |
| **Preparing**: Getting an organization and its staff ready to use it | Convince clinicians about the validity, reliability and utility of PROMs | Characteristics of individuals: Knowledge and beliefs about the intervention | The PROM underwent an intensive validation process with patient and provider engagement. | Educate: Conduct educational meetings  *The educational meeting will provide data on the psychometric properties of the PROM.* |
|  | Engage clinicians in the process | Process: Engaging (e.g. education, training) | - | Plan: Organize clinician implementation team meetings  *See all strategies aimed to educate.* |
|  | Provide practical training for clinicians | Inner setting: Readiness for implementation -Access to knowledge and information | - | Educate: Conduct educational meetings  *The educational meeting will cover all practical aspects of the PROM process (e.g., administering, interpreting) as well as explain the justification for their use and expected benefits.*  Educate: Conduct ongoing training  Educate: Provide ongoing consultation |
|  | Invest sufficient available resources in supporting the PROM process | Inner setting: Readiness for implementation -Available resources | An award-winning smartphone app and patient portal was adapted to be used to administer the PROM, improving the accessibility and management of PROM data.  To identify ways of addressing issues raised by the PROM, a prior study was conducted with patient and provider involvement (Intervention Pathways Study). | Educate: Conduct educational meetings  *Managing issues raised by the PROM will be addressed in educational meetings, drawing on the results of the Intervention Pathways Study and knowledge of local services.*  Educate: Provide ongoing consultation  *The Application Manager will provide ongoing support, including PROM data quality and systems monitoring.* |
| **Commencing**: Organization starting to use it | Provide clinicians opportunities to test out using the PROM and become confident using it before it is fully rolled out | Intervention characteristics: Trialability  Characteristics of individuals: Self-efficacy | As a pilot study to a more definitive trial, it inherently provides a period of trialability. It is a small-scale test of the intervention that will allow for refinement of the processes involved. | Quality management: Conduct cyclical small tests of change  *See all strategies aimed to educate.* |
|  | Be prepared for issues to arise when starting to use the PROM | Process: Executing | - | Quality management: Conduct cyclical small tests of change  *Building on feedback received from patients and physicians and the Application Manager’s field notes, we will use a rapid-cycle evaluation approach, using the CFIR, to improve implementation.*  Educate: Provide ongoing consultation |
| **Reflecting and developing**: Reflecting on the process and making improvements | Organizations must spend time reflecting and evaluating the implementation process. | Process: Reflecting & evaluating | The pilot trial was designed to use mixed methods (quantitative and qualitative) to evaluate the progress and quality of the PROM intervention’s implementation. | Quality management: Conduct cyclical small tests of change  *Building on feedback received from patients and physicians and the Application Manager’s field notes, we will use a rapid-cycle evaluation approach, using the CFIR, to improve implementation.* |
|  | Ensure open channels of communication with time and space for clinicians to criticize and feedback on the PROM process | Process: Reflecting & evaluating | - | Plan: Organize clinician implementation team meetings |
|  | Implementation leads must consider feedback and use it to develop the process | Process: Reflecting & evaluating | - | Quality management: Conduct cyclical small tests of change  *Building on feedback received from patients and physicians and the Application Manager’s field notes, we will use a rapid-cycle evaluation approach, using the CFIR, to improve implementation.* |
