# Supplementary material for: Implementation of an electronic patient-reported measure of barriers to antiretroviral therapy adherence with the Opal patient portal: Protocol for a mixed method type 3 hybrid pilot study at a large Montreal HIV clinic
Source: PLoS One. 2021 Dec 30;16(12):e0261006. doi: 10.1371/journal.pone.0261006 (PMC8717992; doi:10.1371/journal.pone.0261006)
Supplement: S1 Appendix — (DOCX) [file pone.0261006.s003.docx]

**- Information and Consent Document –**

**The I-Score/OPAL Implementation Pilot Study - Patient**

**Study title:** Electronic capture through the OPAL patient portal of a patient-reported measure of barriers to antiretroviral therapy adherence: a mixed method type 3 hybrid pilot study at a large Montreal HIV clinic

**Funding:** The CIHR Canadian HIV Trials Network (CTN) (CTNPT 039)

**Principal Investigator:** Bertrand Lebouché MD, PhD

1. **Information about the Study**

**INTRODUCTION**

You are being invited to take part in this research study because you were diagnosed with HIV and you are currently on antiretroviral therapy. The study involves the testing of a new questionnaire for HIV care that will be completed through a smartphone application and patient portal called Opal. Opal has been in use at the McGill University Health Centre for 2 years and was recently adapted for HIV care in our unit, the Chronic Viral Illness Service.

This questionnaire collects information on factors that can *interfere* (the "I" in the I-Score name) with your adherence to HIV medication.

Before you decide to take part in the study and sign the Consent Document, take the time to read, understand and carefully think about the following information.

This Consent Document may contain information or words that you do not understand. You should ask the investigator in charge of the study or members of the study staff to answer your questions and explain any word or information that you do not understand.

**Study Objectives:**

The main objective of this study is to collect information on the views of people with HIV and their HIV physicians on the in-clinic use of the questionnaire (the I-Score measure). Its other objectives are to see how the I-Score measure could be better integrated in HIV care, and if it shows promise for improving patient care and health.

Part of this study involves having a small group of people with HIV on antiretroviral therapy fill out the I-Score measure with their smartphones through Opal (the patient portal), prior to meeting with their treating doctor. The doctor will have access to the results. This will be done on 3 occasions during regular clinic visits.

**Inclusion and Exclusion Criteria for the Participants:**

To be eligible to participate, you must…

**Inclusion criteria**

- be aged 18 years or older
- be diagnosed with HIV-1 infection
- be treated with a combination antiretroviral therapy (composed of 2 to 3 different drugs)
- be treated for HIV at the Chronic Viral Illness Service of the McGill University Health Centre
- be able to speak and understand either French or English
- possess a smartphone

You may not participate if you…

**Exclusion criteria**

- are participating in a clinical trial at the time of enrollment in this study, in some cases
- have a cognitive impairment or medical instability that prevents you from participating in the interview
- have insufficient mastery of French or English to participate in the interview and complete the questionnaires
- are co-infected with hepatitis C and are being treated for it or have completed treatment 3 months or less ago
- are co-infected with hepatitis B and are either not treated for it or are being treated for it with a medication other than your anti-HIV medication

**Study Procedures:**

If you agree to participate, you will be among 32 patients who participate in this study.

Your participation will involve completing a sociodemographic questionnaire (time required: 10 minutes). It will ask about your age, sex, education, income, immigration, ethnicity, and sexual orientation. It will also ask about your HIV and use of mobile devices.

Your participation will also mean doing each of the following things 3 times: at baseline (when you begin your participation), at 3 months and at 6 months into the study:

- Attend a regular clinic visit with your treating HIV physician (yet spaced by 3-month intervals)
- Complete the I-Score measure with the Opal smartphone app before each clinic visit (time required: 10 minutes)
- Complete the research questionnaire online after each clinic visit (time required: 10 minutes). This questionnaire will collect your opinion on the I-Score measure and on your level of ART adherence.

At baseline, 3 months and 6 months, your latest HIV viral load result will be collected from your patient file at the clinic.

At 3 months and 6 months of the study, you may also be asked to participate in a one-on-one 45-minute qualitative interview on your experience using the I-Score measure and the Opal smartphone app. With these interviews we want to know how we could improve these tools. The interview time (45 min) does not include ten minutes to present the objectives of the interview at the start and ten minutes at the end to wrap up the interview (total participation time: 1h05). These interviews will be held by a trained interviewer as a teleconference that you can connect to by the Internet or by telephone. It will be audio-recorded so that it can be transcribed. Your identity will be protected during these interviews; your name and your video image will not be shown on the screen.

You will receive training to use the I-Score measure and the Opal app. You will have access to a member of the team, if you need help with these tools or if you have questions.

After you fill out the I-Score measure, you will be able to look over the results. You will then meet with your doctor who will also receive the results. After your appointment, you will be able to complete the research questionnaire on your experience with the I-Score measure and Opal.

For an overview of study procedures see Table 1.

During this study, you will not be exposed to any invasive medical interventions and you will not be asked to take any medications other than those prescribed to you by your doctor.

**Table 1**. Summary of study procedures.

| **Procedure** | **Timeline** | | | |
| --- | --- | --- | --- | --- |
|  | Prior to study start | Study start (baseline) | Month 3 | Month 6 |
| Participate in the screening and consent process | X |  |  |  |
| Receive training on the I-Score measure and Opal | X |  |  |  |
| Complete the I-Score measure via Opal |  | X | X | X |
| Meet with your HIV physician |  | X | X | X |
| Complete the online sociodemographic questionnaire |  | X |  |  |
| Complete the online research questionnaire |  | X | X | X |
| Possibly participate in a qualitative interview |  |  | X | X |
| Receive compensation |  | X | X | X |

**Participant Responsibilities:**

***Selection visit***

If you wish to participate in this study, you will be asked to read, sign and date this consent form. To be sure that you are eligible to participate, you will need to take part in a selection visit with a research doctor or a member of the research personnel who will verify whether or not you are eligible to participate.

**Study Risks:**

It is possible that you may feel uncomfortable answering some interview questions. If you find the information or questions asked to be sensitive, private, or distressing, you do not have to answer those questions. The study team is available to discuss your concerns and/or to refer you to appropriate resources.

**Possible Benefits:**

There are no material advantages to participating in this study. The information obtained from you could, however, help improve the care of other patients living with HIV in the future. More specifically, this study could contribute to the use of a new clinical tool for HIV patient follow-up.

**Confidentiality:**

While you take part in this study, the study investigator and team will collect and take down information about you in a research study file. Only information necessary for the research study will be collected.

The information in your file could include your past and present medical history, information about your daily life and test results from exams and procedures done during this study. Your file could also contain other information, such as your name, sex, date of birth and ethnic origin.

All the information collected about you during the study will remain confidential as the law demands. To protect your privacy, your information will be identified with numbers and or letters. Only the investigator in charge of the study knows the numbers and/or letters that link them to you.

The study investigator will use the study information collected about you for research purposes, only to reach the study goals as they are explained in this Information and Consent Document. Your study information will be kept by the investigator in charge of the study for 7 years from the date of publication.

The study information could be printed in medical journals or shared with other people at scientific meetings, but it will be impossible to identify you.

To make sure the study is being done properly; your research study file as well as your medical record could be checked by a person authorized by:

- A representative of the Research Ethics Board who may also contact you to ask about your experience as research participant;
- The Research Institute of McGill University Health Centre;

For your safety and to be able to reach you quickly, your family name, first name, coordinates and the date you started and ended the study will be kept for one year after the study ends in a separate list kept by the investigator in charge of the study or by the MUHC.

You have the right to look at your study file to check the information gathered about you and to correct it, if necessary, as long as the study investigator or MUHC keeps this information. However, you may only have access to certain information once the study has ended.

**Voluntary Participation and the Right to Withdraw:**

You may choose whether you would like to take part in this study. If you choose to take part now, you can change your mind later and stop at any time and for any reason. Tell the investigator in charge of the study or one of the members of the research team about your decision. Your participation in this study is entirely voluntary. If you choose to take part now, you can change your mind later and stop at any time and for any reason. Your future medical care and your relationship with your doctor and or other people involved in your care will not change in any way. Tell the investigator in charge of the study or one of the members of the research team about your decision. He or she will explain the best way for you to stop taking part.

The investigator in charge of the study or the Research Ethics Board of may take you off the study without your consent at any time if:

- New information shows that taking part in the study is not right for you;
- You do not follow directions given to you by the investigator in charge of the study or by a member of the study staff.

If you choose to stop taking part or are taken off the study, the information that was already collected from you during the study will be stored as long as legally required.

If information becomes available during the study that may be relevant to your willingness to continue participating, the principal investigator or research staff will inform you in a timely manner.

**Compensation for Participation:**

You will receive $20 each time you complete the online research questionnaire, that is: at baseline, at 3 months and at 6 months into the study, for a total of $60. If you are invited to participate in a one-on-one interview, you will be compensated $30 for each interview you participate in. If you choose to stop taking part in the study or are removed from it before the study is completed, you will be paid only part of this money depending on the length of time you took part.

**Compensation in Case of Injury or Loss and the Rights of the Research Participant:**

If you suffer any injury due to study procedure, you will receive all the care and services needed to treat you without any cost to you.

By accepting to take part in this study, you keep your legal rights and you do not free investigators or the MUHC of any of their civil and professional responsibility toward you.

**Control of the Ethical Aspects of the Research Study:**

The Research Ethics Board of the MUHC approved this study and is responsible for following the study and making sure that you are protected. Before any change is made to the Consent Document or to the study, it must first be approved by the Research Ethics Board.

**Funding of the Study:**

This study will be led by Dr. Bertrand Lebouché and funded by the CIHR Canadian HIV Trials Network (CTN). Be aware that study site doctors receive no direct financial compensation for enrolling you in this study.

**Contact Information:**

If you have any questions concerning matters related to this study, you may contact Dr. Bertrand Lebouché at (514) 843-2090.

Designated research staff member: If you have any questions concerning matters related to this study, you may contact [name, telephone number, institutional email to be determined]

For any question concerning your rights as a research participant taking part in this study or if you have comments, or wish to file a complaint, you may communicate with the Patient Ombudsman of the McGill University Health Centre at the following phone number: 514-934-1934, ext. 35655.

**B. DECLARATION OF CONSENT**

**PARTICIPANT’S CONSENT**

**Study title:** Electronic capture through the Opal patient portal of a patient-reported measure of barriers to antiretroviral therapy adherence: a mixed method type 3 hybrid pilot study at a large Montreal HIV clinic

I have read all **6 pages** and all of my questions have been answered to my satisfaction. I am free to ask further questions at any time during the study, and I will receive a copy of this consent form for my records.

All personal data collected will remain confidential, to the extent possible by applicable laws and regulations, and any resulting publication will maintain my anonymity.

My participation in the study is voluntary and I am completely free to refuse to participate in any aspect or to fully withdraw from this study at any time without this changing in any way the quality of care that I will receive.

I am not waiving any of my legal rights nor am I freeing the investigators, sponsors or the health establishment from their legal and professional responsibilities.

There is no guarantee that this study will provide any benefit to me.

The potential benefits, risks and procedures associated with this study have been fully explained to me and I have had ample time and opportunity to ask questions and to decide whether or not to participate in this study.

I asked that the consent form be provided in English.

| Research participant name (printed) | Research participant signature | Date of consent  (dd mmm yyyy) |
| --- | --- | --- |
|  |  |  |

**Documentation of Consent**

**Person(s) who conducted the study and consent discussion:**

The potential benefits, risks and procedures associated with this study have been fully explained to the volunteering participant and he or she has had ample time and opportunity to ask questions and to decide whether or not to participate in this study.

| Name of person who obtained consent (printed) | Study role of the person who obtained consent | |
| --- | --- | --- |
|  |  | |
| Signature | Date of consent (dd mmm yyyy) |  |
|  |  |  |
| Name of the impartial witness (printed) | Occupation of the impartial witness |  |
|  |  |  |
| Signature | Date of consent (dd mmm yyyy) |  |
|  |  |  |
