# Supplementary material for: Implementation of an electronic patient-reported measure of barriers to antiretroviral therapy adherence with the Opal patient portal: Protocol for a mixed method type 3 hybrid pilot study at a large Montreal HIV clinic
Source: PLoS One. 2021 Dec 30;16(12):e0261006. doi: 10.1371/journal.pone.0261006 (PMC8717992; doi:10.1371/journal.pone.0261006)
Supplement: S2 Appendix — (DOCX) [file pone.0261006.s004.docx]

### Time 1 questionnaire for people living with HIV

***Your sociodemographic characteristics***

*The following questions concern your sociodemographic characteristics.*

1. What is your year of birth? __________
2. What is your sex?

- Female
- Male
- Trans

1. What is your preferred language?

- French
- English
- Other (please specify): ____________________

1. What is the highest level of education that you have completed?

- Primary
- Secondary (High school)/Professional degree
- College/ Cegep/ Technical degree
- University
- Other (please specify): ____________________ ­

1. What is your annual income ($CAD)?

- Less than $10,000
- $10,000-$19,999
- $20,000-$39,999
- $40,000-$59,999
- $60,000-$79,999
- $80,000-$99,999
- Greater than $100,000

1. Did you immigrate to this country?

- Yes
- No

1. What ethnic group(s) or family background(s) do you identify with? Select all that apply.

- Aboriginal or Indigenous
- English Canadian
- French Canadian
- French
- British
- Other Easter/Western European
- East Asian
- South Asian
- West Asian
- Arab or North African
- Latin American
- African
- Black
- Caribbean
- Pacific
- Mixed race/ethnicity
- Other (please specify): ____________________

1. Which of the following best describes your sexual orientation?

- Heterosexual
- Homosexual
- Bisexual

***Your HIV diagnosis and medication***

*The next two questions concern your HIV diagnosis and HIV medication.*

1. What year were you diagnosed with HIV? __________
2. How would you rate your overall satisfaction with your current HIV medication?
   - 1—very dissatisfied
   - 2
   - 3
   - 4
   - 5—very satisfied

***Your use of mobile devices***

*These next questions concern your use of mobile devices.*

1. With what frequency do you use mobile devices (smartphone, tablet computer, cell phone, smartwatch, pocket PC, etc.)?

- Several times a day
- Once a day
- Several times per week
- Several times per month

1. On your tablet or smartphone, do you have any ‘apps’ related to health and wellness?

- Yes
- No

1. To what extent do you use applications related to health on a mobile device?

- Several times a day
- Once a day
- Several times per week
- Several times per month
- Once a month or less
- Never

*Please indicate your level of agreement with the following statements.*

1. I am confident that I can effectively report medical information using mobile technology.

- Strongly agree
- Agree
- Neutral
- Disagree
- Strongly disagree

1. If any health care provider asks me to report personal health data using a mobile device app, I will do so.

- Strongly agree
- Agree
- Neutral
- Disagree
- Strongly disagree

***Your thoughts on using the I-Score***

*In this section, the questions are about your thoughts on using the I-Score measure, as completed through the Opal app.*

1. How easy was the I-Score for you to use?
   - 1—very difficult
   - 2
   - 3
   - 4
   - 5—very easy
2. How understandable were the questions?
   - 1—difficult to understand
   - 2
   - 3
   - 4
   - 5—easy to understand
3. How much did you enjoy using the I-Score?
   - 1—not at all
   - 2
   - 3
   - 4
   - 5—very much
4. How helpful was the I-Score in describing difficulties experienced taking HIV medication?
   - 1—very unhelpful
   - 2
   - 3
   - 4
   - 5—very helpful
   - Not applicable
5. Was the amount of time it took to use the I-Score acceptable?
   - 1—very unacceptable
   - 2
   - 3
   - 4
   - 5—very acceptable
6. How would you rate your overall satisfaction with the I-Score?
   - 1—very dissatisfied
   - 2
   - 3
   - 4
   - 5—very satisfied
7. How likely are you to recommend the I-Score?

- Extremely unlikely
- Unlikely
- Neither likely nor unlikely
- Likely
- Extremely likely

| 1. Using the I-Score seems… | Completely disagree | Disagree | Neither agree nor disagree | Agree | Completely agree |
| --- | --- | --- | --- | --- | --- |
| a. fitting | ➀ | ➁ | ➂ | ➃ | ➄ |
| b. suitable | ➀ | ➁ | ➂ | ➃ | ➄ |
| c. applicable | ➀ | ➁ | ➂ | ➃ | ➄ |
| d. like a good match | ➀ | ➁ | ➂ | ➃ | ➄ |
| e. implementable | ➀ | ➁ | ➂ | ➃ | ➄ |
| f. possible | ➀ | ➁ | ➂ | ➃ | ➄ |
| g. doable | ➀ | ➁ | ➂ | ➃ | ➄ |
| h. easy | ➀ | ➁ | ➂ | ➃ | ➄ |

***Adherence to your HIV medication***

1. Rate your ability to take all your HIV medications as prescribed, over the past 4 weeks.

- Very poor
- Poor
- Fair
- Good
- Very good
- Excellent

**Comments? ______________________________________________________________________________________________________________________________________________________________________________________________________**

***Thank you!***

### Time 1 questionnaire for HIV physicians

***Your sociodemographic characteristics and HIV practice***

*The following five questions concern your sociodemographic characteristics and HIV practice.*

- - 1. What is your year of birth? __________
    2. What is your sex?
- Female
- Male
  - 1. What is your preferred language?
- French
- English
- Other (please specify): ____________________
  - 1. How long have you been treating people with HIV?
- 1-4 years
- 5-9 years
- 10-14 years
- 15-19 years
- 20 years or more
  - 1. Approximately how many people with HIV are you currently following? __________

***Your thoughts on using the I-Score***

*These last questions are about your thoughts on using the I-Score measure, as accessed by you through the ORMS dashboard.*

- - 1. How easy was the I-Score for you to use?
  - 1—very difficult
  - 2
  - 3
  - 4
  - 5—very easy

1. How understandable were the questions?
   - 1—difficult to understand
   - 2
   - 3
   - 4
   - 5—easy to understand
2. How much did you enjoy using the I-Score?
   - 1—not at all
   - 2
   - 3
   - 4
   - 5—very much
3. How helpful was the I-Score in describing difficulties experienced taking HIV medication?
   - 1—very unhelpful
   - 2
   - 3
   - 4
   - 5—very helpful
   - Not applicable
4. Was the amount of time it took to use the I-Score acceptable?
   - 1—very unacceptable
   - 2
   - 3
   - 4
   - 5—very acceptable
5. How would you rate your overall satisfaction with the I-Score?
   - 1—very dissatisfied
   - 2
   - 3
   - 4
   - 5—very satisfied
6. How likely are you to recommend the I-Score?

- Extremely unlikely
- Unlikely
- Neither likely nor unlikely
- Likely
- Extremely likely

| 1. Using the I-Score seems… | Completely disagree | Disagree | Neither agree nor disagree | Agree | Completely agree |
| --- | --- | --- | --- | --- | --- |
| a. fitting | ➀ | ➁ | ➂ | ➃ | ➄ |
| b. suitable | ➀ | ➁ | ➂ | ➃ | ➄ |
| c. applicable | ➀ | ➁ | ➂ | ➃ | ➄ |
| d. like a good match | ➀ | ➁ | ➂ | ➃ | ➄ |
| e. implementable | ➀ | ➁ | ➂ | ➃ | ➄ |
| f. possible | ➀ | ➁ | ➂ | ➃ | ➄ |
| g. doable | ➀ | ➁ | ➂ | ➃ | ➄ |
| h. easy | ➀ | ➁ | ➂ | ➃ | ➄ |

1. Using the I-Score is compatible with all aspects of my work

- Extremely disagree
- Disagree
- Somewhat disagree
- Neither agree nor disagree
- Somewhat agree
- Agree
- Extremely agree

1. Using the I-Score is completely compatible with my current work situation

- Extremely disagree
- Disagree
- Somewhat disagree
- Neither agree nor disagree
- Somewhat agree
- Agree
- Extremely agree

1. I think that using the I-Score fits well with the way I like to work

- Extremely disagree
- Disagree
- Somewhat disagree
- Neither agree nor disagree
- Somewhat agree
- Agree
- Extremely agree

1. Using the I-Score fits into my work style

- Extremely disagree
- Disagree
- Somewhat disagree
- Neither agree nor disagree
- Somewhat agree
- Agree
- Extremely agree

**Comments? ______________________________________________________________________________________________________________________________________________________________________________________________________**

***Thank you!***
