# Supplementary material for: Implementation of an electronic patient-reported measure of barriers to antiretroviral therapy adherence with the Opal patient portal: Protocol for a mixed method type 3 hybrid pilot study at a large Montreal HIV clinic
Source: PLoS One. 2021 Dec 30;16(12):e0261006. doi: 10.1371/journal.pone.0261006 (PMC8717992; doi:10.1371/journal.pone.0261006)
Supplement: S1 Dataset — (DOCX) [file pone.0261006.s005.docx]

**World Health Organization Trial Registration Data Set for study CTNPT039**

| Data category | Information |
| --- | --- |
| 1 **Primary Registry and Trial Identifying Number** | ClinicalTrials.gov  Identifier: NCT04702412 |
| 2 **Date of Registration in Primary Registry** | January 8, 2021 |
| 3 **Secondary Identifying Numbers** | Canadian HIV Trials Network (CTN)  CTNPT039 |
| 4 **Source(s) of Monetary or Material Support** | Canadian HIV Trials Network  Merck Canada Inc. |
| 5 **Primary Sponsor** | Research Institute of the McGill University Health Centre |
| 6 **Secondary Sponsor(s)** | n.a. |
| 7 **Contact for Public Queries** | Bertrand Lebouché, MD, PhD [Bertrand.lebouche@mcgill.ca](mailto:Bertrand.lebouche@mcgill.ca),  Postal address: McGill University Health Centre, Chronic Viral Illness Service, Royal Victoria Hospital, D02.4110 - Glen Site, 1001 boulevard Decarie Montreal (Québec) Canada, H4A 3J1.  Tel: +1 514-843-2090 |
| 8 **Contact for Scientific Queries** | Principal investigator Bertrand Lebouché, MD, PhD [Bertrand.lebouche@mcgill.ca](mailto:Bertrand.lebouche@mcgill.ca),  Postal address: McGill University Health Centre, Chronic Viral Illness Service, Royal Victoria Hospital, D02.4110 - Glen Site, 1001 boulevard Decarie Montreal (Québec) Canada, H4A 3J1.  Tel: +1 514-843-2090  Co-Principal investigator Kim Engler, PhD [kimcengler@gmail.com](mailto:kimcengler@gmail.com) Postal address: McGill University Health Centre, Chronic Viral Illness Service, Royal Victoria Hospital, D02.4110 - Glen Site, 1001 boulevard Decarie Montreal (Québec) Canada, H4A 3J1.  Tel: +1 514-934-1934 x-32126 |
| 9 **Public Title** | Electronic Capture of Adherence Barriers for HIV Care: A Pilot Study Protocol |
| 10 **Scientific Title** | Implementation of an Electronic Patient-Reported Measure of Barriers to Antiretroviral Therapy Adherence with the Opal Patient Portal: Protocol for a Mixed Method Type 3 Hybrid Pilot Study at a Large Montreal HIV Clinic |
| 11 **Countries of Recruitment** | Canada |
| 12 **Health Condition(s) or Problem(s) Studied** | HIV, adherence to antiretroviral therapy |
| 13 **Intervention(s)** | Patient use of a smartphone app (a patient portal called Opal) to routinely complete a new measure of barriers to antiretroviral therapy adherence and report these results electronically to the treating HIV physician. |
| 14 Key Inclusion and Exclusion Criteria | Confirmed HIV positive  Aged at least 18 years old  On combination ART Literate in English or French  Own a smartphone  Willing to download the smartphone app. Known or suspected adherence problems in the past 12 months  Exclusion:  Concurrent enrolment in a clinical trial Cognitive impairment or medical instability Insufficient ability to use the app with the technical support provided |
| 15 Study Type | Interventional Intervention model: single group assignment Design: Pilot study with a mixed method type 3 implementation-effectiveness hybrid design Primary purpose: of study -Evaluate participant perceptions of the intervention and implementation strategy to plan a definitive trial; of intervention -Screening |
| 16 **Date of First Enrollment** | Anticipated: July 2021 |
| 17 **Sample Size** | 32 patients |
| 18 **Recruitment Status** | Not yet recruiting |
| 19 **Primary Outcome(s)** | Outcome Name: Acceptability of the Intervention Metric/method of measurement: Acceptability E-Scale, Percent likely to recommend the I-Score, and the Net Promoter Score Timepoint: Change from baseline to week 24 |
| 20 **Key Secondary Outcomes** | Outcome Name: Appropriateness of the Intervention  Metric/method of measurement: Perceived Compatibility subscale and Appropriateness of Intervention Measure  Timepoint: Change from baseline to week 24  Outcome Name: Feasibility of the Intervention  Metric/method of measurement: Consent rate, Retention rate, Missing I-Score data rate, Feasibility of Intervention Measure  Timepoint: Baseline, Cumulative until week 24, Cumulative until week 24, Change from baseline to week 24, respectively.  Outcome Name: Fidelity  Metric/method of measurement: Percent patients who completed the I-Score on time, Percent physicians who reviewed the I-Score results on time.  Timepoint: Cumulative until week 24. |
| 21 **Ethics Review** | Status: Approved  Date: January 18, 2021  Ethics committee: The Cells, Tissues, Genetics & Qualitative research panel of the McGill University Health Centre Research Ethics Board (Study code: CTNPT039/2021-7190). |
| 22 **Completion date** | Anticipated: April 2022 |
| 23 **Summary Results** | n.a. |
| 24 **IPD sharing statement** | Plan to share IPD -Yes, following study completion. |
