# Supplementary material for: Implementation of an electronic patient-reported measure of barriers to antiretroviral therapy adherence with the Opal patient portal: Protocol for a mixed method type 3 hybrid pilot study at a large Montreal HIV clinic
Source: PLoS One. 2021 Dec 30;16(12):e0261006. doi: 10.1371/journal.pone.0261006 (PMC8717992; doi:10.1371/journal.pone.0261006)
Supplement: S1 File — (PDF) [file pone.0261006.s006.pdf]

The I-Score/Opal Implementation Pilot Study

Implementation of an electronic patient-reported measure of barriers to antiretroviral therapy adherence with the Opal patient portal: a mixed method type 3 hybrid pilot study at a large Montreal HIV clinic

Protocol

Confidentiality Statement

The confidential information in this document is provided to you, as an Investigator or consultant, for review by you, your staff, or a Research Ethics Board.

Good Clinical Practice Statement

This study will be conducted in accordance with the applicable Health Canada regulations, International Conference on Harmonisation (ICH) guidelines on current Good Clinical Practice (GCP), and the Declaration of Helsinki.

Version: 16December , 2020

Table of Contents

Participating site....4

Scientific committee....4

Research personnel....5

Collaborators....6

List of terms and abbreviations....6

Summary....7

Introduction....8

- Aim and objectives....8
- Guiding frameworks....8
- Methods....10
  - Study design....10
  - Setting and participants....11
  - Patient eligibility criteria....11
  - Recruitment and consent process....12
  - The I-Score intervention....12
- Data collection....13
  - Quantitative component....13
  - Qualitative component....14
- Study metrics and instruments....14
  - Objective 1 -Evaluate perceptions of the I-Score intervention....15
  - Objective 2 -Evaluate the implementation strategy....16
  - Objective 3 -Determine preliminary intervention and effectiveness outcome utility....16
- Data analysis....16
  - Quantitative analysis....16
  - Qualitative analysis....17
  - Mixed methods analysis....18
- Participant security....18
  - Adverse events (AE's)....18
- Expected study contributions....18
  - Scientific contributions....19
- Communication of results and knowledge transfer....19
  - Scientific....19

Clinical 19
Community 19
Publication policy 19
Project timeline 19
Anticipated problems 19
Project management 20
Scientific committee 20
Research personnel 20
Collaborators 20
Ethical considerations 20
Confidentiality 20
Obtaining consent 21
Ethics approval 21
Good clinical practices statement 21
Protocol deviations and violations 21
Source documents 21
Relevant skills of the scientific committee members 21
Budget 22
References 23
Appendix 32
Physician email invitation 33
Refusal form 34
Time 1 questionnaire for people living with HIV 35
Time 1 questionnaire for HIV physicians 39
Time 2 and Time 3 questionnaire for people living with HIV 42
Time 2 and Time 3 questionnaire for HIV physicians 44
Physician checklist per HIV patient visit 47
Qualitative interview schedule 1 (Time 2) 48
Qualitative interview schedule 2 (Time 3) 49
Investigator signature page 50

Figure 1. Guiding implementation framework and I-Score intervention logic chain. 9
Figure 2. Relationship between the I-Score implementation strategy and study outcomes. 10
Figure 3. Pilot study design. 11

Table 1. Schedule of study procedures for participants 14

Table 2. Relationship between the identified facilitators/barriers of PROM implementation, the implementation framework (CFIR), and the pilot study's implementation strategies. 27
Table 3. Implementation science metrics and effectiveness outcomes collected for the pilot study. 31

Participating site

Funding CIHR Canadian HIV Trials Network (Study CTNPT 039)

Principal investigator Bertrand Lebouché MD, PhD, Associate Professor, Department of Family Medicine, McGill University; Research Institute, McGill University Health Centre (MUHC); Chronic Viral Illness Service, Royal Victoria Hospital, 1001 Decarie Blvd., D02.4017, Montreal, Quebec, H4A 3J1, Canada. Tel: 1 (514) 823-7587; Fax: 1 (514) 843-2092.

Chronic Viral Illness Service Royal Victoria Hospital – Glen Site, McGill University Health Centre (MUHC), 1001 boul. Décarie., D02.4017, Montréal, Québec, H4A 3J1

Scientific committee

Name and title(s)	Coordinates	Responsibilities
Bertrand, Lebouché, MD, PhD	Chronic Viral Illness Service<br>Royal Victoria Hospital<br>1001 Decarie Blvd., D02.4017<br>Montreal, Quebec, H4A 3J1<br>Tel: 514-823-7587<br>Fax: 514-843-2092<br>bertrand.lebouche@mcgill.ca	Principal investigator
Kim Engler, PhD	Chronic Viral Illness Service<br>Royal Victoria Hospital<br>1001 Decarie Blvd., D02.4017<br>Montreal, Quebec, H4A 3J1<br>Tel: 514-934-1934 ext 32126<br>Fax: 514-843-2092<br>kimcengler@gmail.com	Co-principal investigator
Tibor Schuster, PhD	Department of Family Medicine, McGill University<br>tibor.schuster@mcgill.ca	Co-investigator
John Kildea, PhD	Medical Physics Unit, McGill University<br>Department of Oncology, McGill University<br>Cedars Cancer Centre, Medical Physics, DS1.7141<br>McGill University Health Centre - Glen Site<br>1001 boul. Décarie<br>Montréal, QC H4A 3J1<br>Tel: 514 934 1934 ext 44154<br>john.kildea@mcgill.ca	Co-investigator
Tarek Hijal, MD	McGill University Health Centre<br>Department of Radiation Oncology<br>Cedars Cancer Centre<br>1001 Decarie Boulevard<br>Montreal, Quebec H4A 3J1<br>Tel: 514-934-4400	Co-investigator

	Tarek.hijal@mcgill.ca	
Joseph Cox, MD	Chronic Viral Illness Service<br>Royal Victoria Hospital<br>1001 Decarie Blvd., D02.4017<br>Montreal, Quebec, H4A 3J1<br>Tel: 514-843-2090<br>Tarek Hijal Fax: 514-843-2092<br>joseph.cox@mcgill.ca	Co-investigator
Sara Ahmed, PhD	Faculty of Medicine, School of Physical & Occupational Therapy<br>McGill University Health Center, Clinical Epidemiology<br>sara.ahmed@mcgill.ca	Co-investigator
Nitika Pant Pai, MD	Division of Clinical Epidemiology – Department of Medicine, McGill University<br>Scientist, Infectious Diseases and Immunity in Global Health Program<br>5252 De Maisonneuve West, #3D.56<br>Montreal, QC H4A 3S9<br>Tel: 514-934-1934 ext 44729<br>nitika.pai@mcgill.ca	Co-investigator
Marina Klein, MD	Chronic Viral Illness Service<br>Royal Victoria Hospital<br>1001 Decarie Blvd., D02.4017<br>Montreal, Quebec, H4A 3J1<br>Tel: 514-843-2090<br>Fax: 514-843-2092<br>marina.klein@mcgill.ca	Co-investigator
Alexandra de Pokomandy, MD	Chronic Viral Illness Service<br>Royal Victoria Hospital<br>1001 Decarie Blvd., D02.4017<br>Montreal, Quebec, H4A 3J1<br>Tel: 514-843-2090<br>Fax: 514-843-2092<br>alexandra.depokomandy@mcgill.ca	Co-investigator
Sofiane Achiche, PhD	École Polytechnique de Montréal<br>Département de Génie Mécanique<br>C.P. 6079, succ. Centre-Ville, Montréal<br>Québec, H3J 3A7, Canada<br>Tel : 514-340-4711 ext 4317<br>sofiane.achiche@polymtl.ca	Co-investigator

Research personnel

Name and title(s)	Coordinates	Responsibilities
David Lessard, PhD	Chronic Viral Illness Service,<br>Royal Victoria Hospital<br>1001 Decarie Blvd., D02.4017	Research assistant

	Montreal, Quebec, H4A 3J1<br>Tel: 514-934-1934 ext 32126<br>Fax: 514-843-2092<br>david.lessard2@mail.mcgill.ca	
Adriana Rodriguez, PhD	Chronic Viral Illness Service,<br>Research Institute of the MUHC<br>1001 Decarie Blvd.,<br>Montreal, Quebec, H4A 3J1<br>Tel: 438-822-2004<br>Fax: 514-843-2092<br>adriana.rodriguez@muhc.mcgill.ca	Research coordinator
Yuanchao Ma, M.Sc.A	Chronic Viral Illness Service,<br>Royal Victoria Hospital<br>1001 Decarie Blvd., D02.4017<br>Montreal, Quebec, H4A 3J1<br>Tel: 514-934-1934 ext 32126<br>Fax: 514-843-2092<br>yuanchao.ma@muhc.mcgill.ca	Application Manager
Serge Vicente, PhD (c)	Chronic Viral Illness Service,<br>Royal Victoria Hospital<br>1001 Decarie Blvd., D02.4017<br>Montreal, Quebec, H4A 3J1<br>Tel: 514-934-1934 ext 32126<br>Fax: 514-843-2092<br>sergevicente1975@gmail.com	Data analyst,<br>Postdoctoral fellow

Collaborators

Name and title(s)	Coordinates	Responsibilities
I-Score Consulting Team	Research Institute, McGill University Health Centre	Provide patient expertise and feedback on the study and participate in knowledge translation
Patrick Keeler	info@cercleorange.ca<br>Le Cercle Orange -cost-free referral and support service for people living with HIV in Montreal without access to health care, COCQ-SIDA	Provide community-based expertise and feedback on the study

List of terms and abbreviations

Term/abbreviation	Definition
ART	Antiretroviral therapy
GCP	Good clinical practice
HIV	Human immunodeficiency virus
I-Score	(ART) Interference-Score
MUHC	McGill University Health Centre
PROM	Patient-reported outcome measure

Summary

Title: Electronic capture through the Opal patient portal of a patient-reported measure of barriers to antiretroviral therapy adherence: a mixed method type 3 hybrid pilot study at a large Montreal HIV clinic

Short title: The I-Score/Opal Implementation Pilot Study

Background: Many people with HIV on antiretroviral therapy (ART) have trouble adhering to their treatment. Regular monitoring of barriers to ART adherence is clinically recommended, however, patient-provider communication around adherence is often inadequate. Our team thus decided to develop a new electronically administered patient-reported measure of barriers to ART adherence (the I-Score) to facilitate the systematic capture of this data for physician consideration in routine HIV care. To develop the tools and methods necessary for a controlled definitive trial to test the I-Score intervention, a pilot study was in order.

Objectives: The specific primary objectives of this pilot study are to evaluate perceptions of the I-Score intervention (Objective 1) and evaluate its implementation strategy (Objective 2). Its secondary objective (Objective 3) is to determine if the intervention shows promise and if the chosen outcomes are useful.

Methods: This 6-month study will adopt a one-arm mixed method type 3 implementation-effectiveness hybrid design and be conducted in a single clinical site, the Chronic Viral Illness Service of the McGill University Health Centre. It will be guided by tools of implementation science (e.g., frameworks, outcomes). Five HIV physicians and 30 of their HIV patients will participate. The intervention will involve having patients complete the I-Score with an adapted version of the Opal smartphone app, prior to meeting with their physician. Both the patient and the physician will have access to the I-Score results, for consideration during the clinic visits at Time 1 (T1), T2 (3 months), T3 (6 months). The implementation strategy will focus on patient and provider involvement, education, and training, as well as on promoting the intervention's adaptability. The strategy's cornerstone will be the hiring of an Application Manager to facilitate implementation. Implementation, patient, and service outcomes will be collected (T1, T2, T3). The primary outcome of this pilot study is the intervention's acceptability (target: mean scale score of 4 on 5). Qualitative data obtained through provider focus groups (T2, T3), patient interviews (T2, T3) and the Application Manager's field notes (T1-3) will serve especially to evaluate the implementation strategy and inform any methodological adaptations.

Timeline:

- January-February 2021: Physician and patient enrollment
- March 2021-August 2021: Data collection
- April 2021-October 2021: Data analysis
- September 2020-December 2021: Presentation and manuscript preparation (beginning with the protocol manuscript)

Expected contributions: This study will contribute data towards the planning of a definitive trial to test the efficacy of the I-Score intervention. Furthermore, it will generate rare data on electronic PROM interventions in routine HIV care that will help improve understanding of conditions for their successful implementation. It could also eventually lead to the implementation of a new tool for HIV care that could shift the HIV care paradigm toward a more patient-centered and preventative approach.

Introduction

Routinely collecting data on patient-reported outcome measures (PROMs) for individual patient care may benefit both people living with HIV and their providers, yet it is seldom done in HIV clinical practice (Kall et al., 2020). For patients, it may help ensure that HIV care is person-centered and in line with their needs (Kall et al., 2020). For providers, given the multidimensional and chronic nature of HIV clinical assessment and follow-up, the use of PROMs could facilitate efficient application of clinical guidelines in a context of time and resource constraints (Kjær et al., 2018).

The management of antiretroviral therapy (ART) adherence for the treatment of HIV provides a case in point. Successful ART remains essential to a near-normal life expectancy; however, many on ART have suboptimal adherence (Bezabhe et al., 2016, Ortego et al., 2011), even on single-tablet regimens (Cohen et al., 2020). In a recent study, only 23% percent of adults initiating a single-tablet regimen were considered adherent over a six-month period versus 12% among those who initiated a multiple tablet regimen, based on prescription fill dates (Cohen et al., 2020). Clinically recommended strategies to foster adherence include ongoing monitoring of barriers to adherence among people living with HIV (DHHS, 2019). Yet, several studies point to inadequate patient-provider communication around ART adherence and its impediments (e.g., Barfod et al., 2006; Beach et al., 2015; Malta et al., 2005; Laws et al., 2013; Wilson et al., 2010) and many HIV providers underestimate their patients' adherence difficulties (Fredericksen et al., 2012; Miller et al., 2002). In addition, individuals with HIV collectively report a multitude of barriers to adherence, including a variety of cognitive, emotional, social and material issues as well as health service-related barriers (Engler et al., 2018; Shubber et al., 2016), the proper evaluation of which may be time-consuming for providers (Genberg et al., 2015).

For these reasons, with patient (Lessard et al., 2019) and provider (Toupin et al., 2018) involvement, we are developing a PROM of barriers to ART adherence, the Interference-Score (or I-Score), for electronic administration. I-Score data will be collected from patients and fed back to their providers via Opal, a patient portal and smartphone app. This award-winning app (Kildea et al., 2019), which is currently in use at the Cedars Cancer Centre of the McGill University Health Centre (MUHC), will be configured to respond to the needs of patients with HIV. Opal gives patients access to their appointment schedules, laboratory test results, educational material, waiting room management tools, and PROMs. Electronic administration of our PROM was crucial as it simplifies score integration within the clinical workflow, allows for longitudinal presentation of scores as well as remote monitoring, and through Opal, it provides access to several useful and potentially empowering patient-centered functions.

Aim and objectives

With the present mixed method pilot study, drawing on implementation science, the aim is to develop the methods and tools necessary to undertake a more robust evaluation of the implementation and effectiveness of the I-Score ePROM-within-Opal innovation (henceforth, the I-Score intervention) in routine HIV care with individuals on ART. This study's primary objectives are to evaluate perceptions of the I-Score innovation (Objective 1) and evaluate the implementation strategy (Objective 2) in terms of recommended implementation science metrics for patient-reported outcome measure initiatives in routine care (Stover et al., 2020). Its secondary objective (Objective 3) is to determine if the intervention shows promise and the chosen outcomes are useful, by observing collected data on select effectiveness outcomes (patient and service outcomes).

Guiding frameworks

It is important that a credible causal explanation of a digital health innovation's intended impacts be provided (Murray et al., 2016). Indeed, in an ePROM-based intervention, conceptual or theoretical frameworks specify the mechanisms through which the intervention is expected to have its effects (Greenhalgh et al., 2017), facilitating appropriate outcome selection and the interpretation of results (Greenhalgh et al., 2005).

This pilot study will be guided, in part, by an intervention logic chain, depicted by the boxes in Figure 1, adapted from the frameworks of Greenhalgh and colleagues (2005; 2017). The core of the intervention is encompassed by the dashed line. The left arrow presents the key components of the implementation framework used, which will guide qualitative analysis. Specifically, these are the five broad domains of potential influence on implementation of Damschroder and colleagues' (2009) Consolidated Framework for Implementation Research (CFIR) within which are grouped 39 distinct constructs. Hence, it is assumed that flow through the logic chain will be affected by features of the intervention, settings, individuals, and implementation process involved. The CFIR is a flexible and widely used framework in implementation research, including for PROM-based initiatives (Stover et al., 2020). Asterisks indicate elements of the logic chain which will be examined as a part of this pilot study.

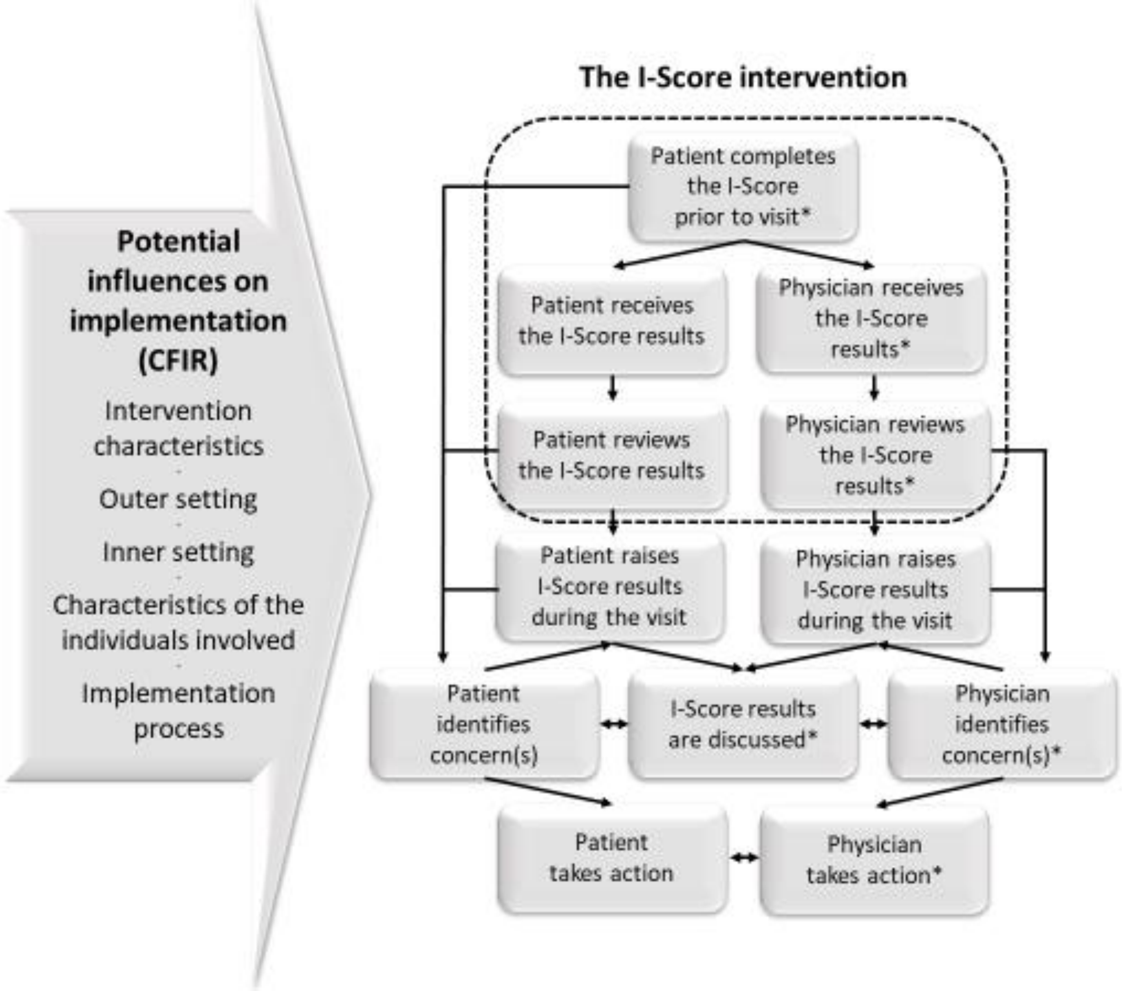

Figure 1. Guiding implementation framework and I-Score intervention logic chain.

Figure 2 is a framework, in progress, that presents the broad hypothesized relationships between the implementation strategy used for the I-Score and the categories of study outcomes addressed. Borrowing from the frameworks of Stover and colleagues (2020) and Santana and Feeny (2014), it, in part, conceives successful implementation of I-Score use in standard HIV care, as potentially generating cascading effects on service and patient outcomes. Asterisks indicate outcomes for which data will be collected as a part of this study.

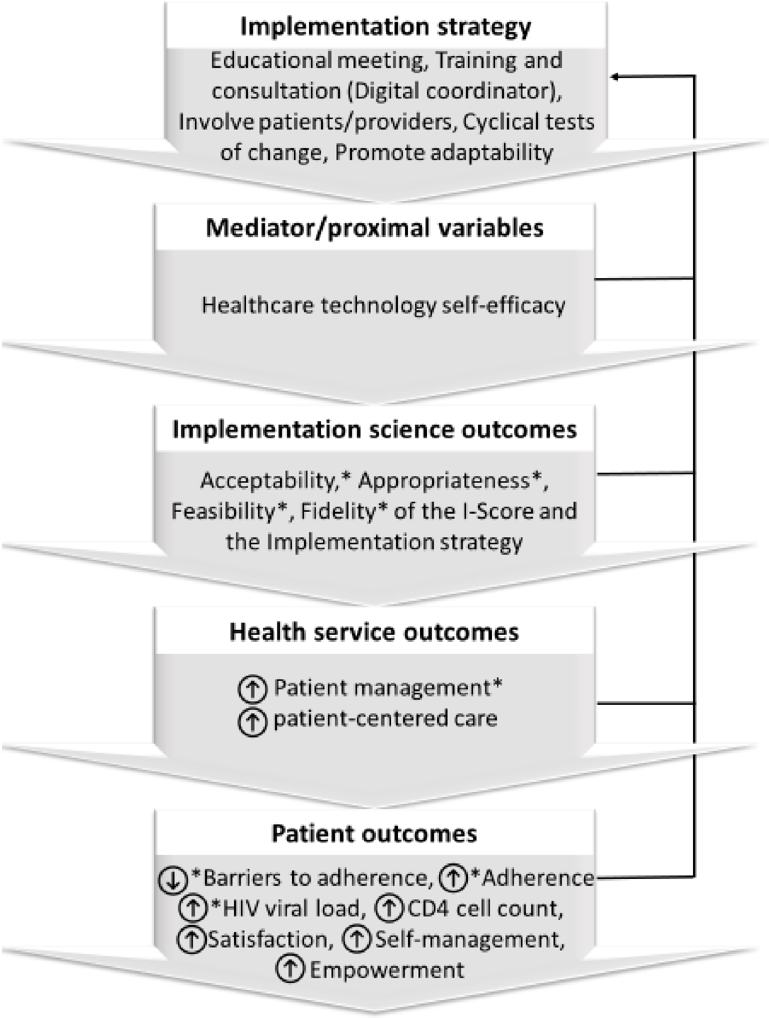

Figure 2. Relationship between the I-Score implementation strategy and study outcomes.

Methods

Study design

This 6-month pilot study will adopt a one-arm mixed method type 3 implementation-effectiveness hybrid design and be conducted in a single clinical site (see Figure 3). Type 3 hybrid designs emphasize testing the implementation strategy of an evidence-based intervention, but merely observing data collected on intervention effectiveness (Curran et al., 2012). While syntheses of effectiveness evidence for PROM use in routine care have typically found mixed results, with inconsistent impacts on patient outcomes (e.g., Boyce et al., 2013, Valderas et al., 2008), a more recent systematic review finds the evidence supports PROM use in standard care, particularly to improve patient-provider communication and decision-making in clinical practice (Isaque et al., 2019). The hybrid design also seems justified, given international momentum for PROM use (Rutherford et al., 2020), which may build with the COVID-19 pandemic. Indeed, there are calls for a scale up of electronic PROMs implementation in this crisis for the remote follow-up of chronic conditions, in part, to better screen patients and promptly manage their needs (Marandino et al., 2020).

Mixed methods were adopted in this study as multiple methods are recommended for studying intervention implementation and its problems in complex systems, like HIV clinics (Peters et al., 2013). Regarding the integration of the quantitative and qualitative data collected, this will occur toward study end within a convergent parallel design (Creswell and Plano Clark, 2011). Those directly involved in the analyses will decide upon the specifications of integration. Reporting of this study will seek to satisfy the standards of Good Reporting of a

Mixed Methods Study (O'Cathain et al., 2008) and the Standards for Reporting Implementation Studies (Pinnock et al. 2017).

Setting and participants

The study setting is a large hospital-based clinic in Montreal, Quebec, Canada. This clinic, the Chronic Viral Illness Service (CVIS) of the MUHC, offers multidisciplinary care to approximately 2000 adults living with HIV. Five physicians treating individuals with HIV at the CVIS will be recruited to participate as well as 30 of their adult patients. This sample size amply meets rule of thumb recommendations for one-arm pilot studies (Sim & Lewis, 2011). Patients must be confirmed HIV-positive, have a history of known or suspected adherence problems in the past year, be aged at least 18 years old, be on or beginning combination ART, be literate in English or French, and own a smartphone. Full details on eligibility are presented in the next section. Ten female patients will be recruited, to ensure sufficient representation of women living with HIV (Public Health Agency of Canada, 2018).

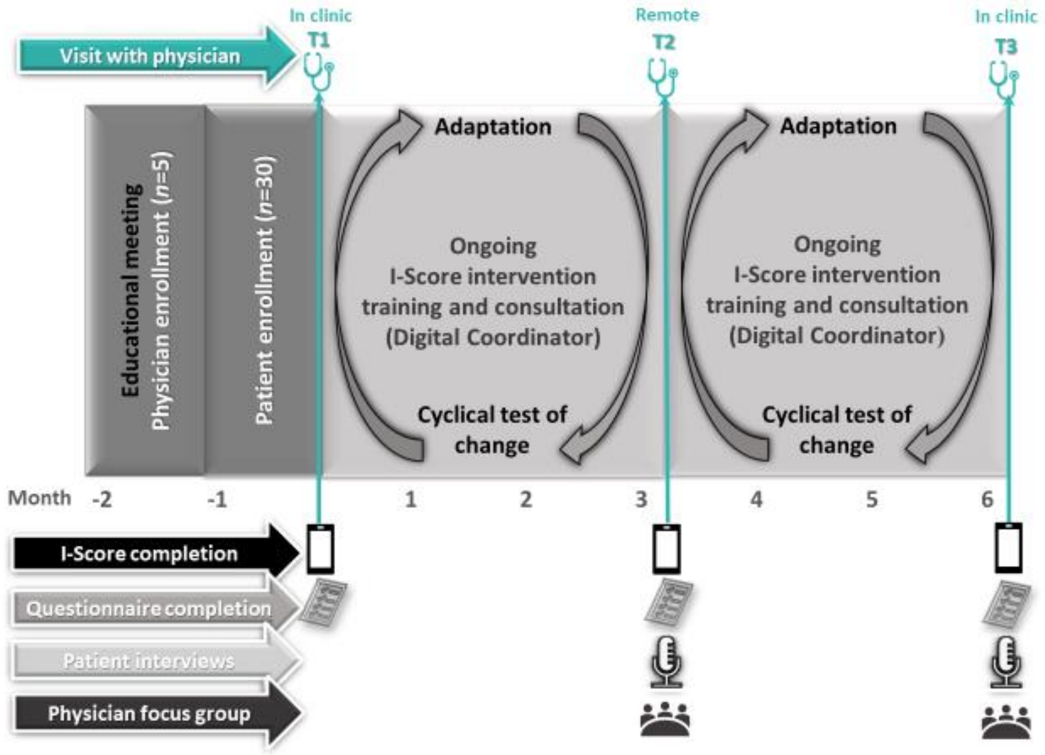

Figure 3. Pilot study design.

Patient eligibility criteria

To be eligible to participate patients must…

Inclusion criteria

- be aged 18 years or older
- be diagnosed with HIV-1 infection
- be treated with a combination antiretroviral therapy (composed of 2 to 3 different drugs)
- have had known or suspected adherence problems in the past 12 months based on a detectable viral load test result per local standards, patient report, and/or report by the healthcare team (by the physician, nurse, social worker or pharmacist)
- be treated for HIV at the Chronic Viral Illness Service of the McGill University Health Centre
- be able to speak and understand either French or English
- possess a smartphone with an appropriate data plan and/or home Wi-Fi connection

- be willing to download the smartphone app

Patients many not participate if they…

Exclusion criteria

- are participating in a clinical trial at the time of enrollment in this study
- have a cognitive impairment or medical instability that prevents them from participating in the interview
- have insufficient mastery of French or English to participate in the interview and complete the questionnaires
- have insufficient ability to use the app with the technical support provided
- are co-infected with hepatitis C and are being treated for it or have completed treatment 3 months or less ago
- are co-infected with hepatitis B and are either not treated for it or are being treated for it with a medication other than their anti-HIV medication

Recruitment and consent process

Physicians treating patients with HIV at the CVIS will be asked individually to participate, by oral or email invitation (see the Appendix for the email invitation). Patients of participating physicians will be recruited and consented in two ways: 1) when they visit the clinic; or 2) prior to an upcoming clinic visit. When they visit the clinic to meet with a health or social service provider (e.g., physician, nurse, social worker, psychologist), the provider will briefly describe the study. Alternatively, patients with upcoming visits will be identified and contacted to inform them of the study. If the individual agrees, the study coordinator will present the project in greater detail to them in person or over the phone, check the eligibility criteria, and obtain consent. Then, an appointment for a teleconference (on Zoom) will be made with the participant prior to their next regular clinic appointment with their physician to deliver training to use the app and complete the I-Score. Given efforts to limit in-hospital visits and risks for patients during the COVID-19 pandemic, in-clinic appointments with research staff will be avoided, when possible.

The I-Score intervention

During the study, participating patients will visit with their HIV physician three times, at Time 1 (T1), month 3 (T2), and month 6 (T3), prior to which they will complete the I-Score, as instructed. This visit schedule was selected as it fits within guidelines for the clinical follow-up of HIV in Quebec (Ministère de la santé et des services sociaux, 2019), while maximizing the collection of repeated study measurements. Given COVID-19, one visit (T2) will be done remotely (by phone or teleconferencing), while the other visits will be held at the CVIS. The I-Score PROM contains 20 items, covering 6 domains of barriers to ART adherence. Respondents indicate how often each barrier made adherence difficult in the past 4 weeks, with a continuous scale, from never to always. [Additional details on measure scoring and interpretation will be provided to the REB as they become available]. For details on the measure's development see Engler et al., 2018, Engler et al., 2019, Lessard et al., 2019, Toupin et al., 2018.

The core intervention of this pilot consists of having individuals with HIV on ART register on the Opal app and complete the I-Score PROM prior to each of three consecutive visits with their HIV physician. The patients will receive a reminder to complete the I-Score one week before their visit and they will have immediate access to their results. The HIV physician will acquire the I-Score results before each visit via the ORMS dashboard, an appointment and questionnaire management tool integrated with Opal and designed specifically for healthcare providers. The option of graphically presenting scores over time will also be available, allowing physicians and patients to compare past and present scores. It is expected that patients and physicians will review the I-Score results so they can be considered during the clinic visit (see Figure 1).

Privacy and security

The technical cybersecurity aspects of the Opal app conform to the security and governance recommendations for

the development of a patient portal, as identified by the MUHC's Security and Governance team, to ensure the confidentiality of patient data (for details, see the multimedia appendix 2 of Kildea et al., 2019).

The implementation strategy

The multilevel ePROM implementation strategy (Curran et al., 2012), designed for this study, addresses known facilitators and barriers to implementing ePROMs in routine clinical practice (Foster et al., 2018) and draws on recognized implementation strategies (Powell et al., 2012, 2015). Table 1 presents the correspondence between the facilitators and barriers targeted, the chosen implementation strategies to address them, and their relationship to components of the implementation framework used in this study, the CFIR. We will conduct an *Educational meeting* by teleconference with providers and other organizational stakeholders to formally teach them about the intervention and its rationale and respond to concerns. We will provide *Training and consultation* on the ePROM and app by hiring an *Application Manager* (AM). The AM will be available to patients and providers on an ongoing basis, as needed, preferentially by phone or teleconference. They will also help monitor the quality of PROM data. Appointing such a coordinator (or Quality Assurance officer) is a recommended strategy to minimize the impact of missing PROM data (Mercieca-Bebber et al., 2016). The AM will thus oversee the completeness of ePROM data collected and manage any system or software problems, which are potential disadvantages of computer or web-based PROM administration (ISOQOL, 2011). Hence, overall, they will facilitate PROM implementation. Another strategy aims to meaningfully *Involve patients and providers* in the I-Score's implementation. Individuals from both groups will provide input on the proposed study prior to its conduct. Following the I-Score administrations at T2 and T3, physicians will participate in a focus group (by teleconference), while a short semi-structured interview will be conducted with each patient (by telephone or teleconference). Throughout the study, the AM will take field notes on the problems encountered by participants and this feedback will enable *Cyclical small tests of change* to improve implementation, using an evaluation approach guided by the CFIR (Consolidated Framework for Implementation Research) (Keith et al., 2017). This way, we will *Promote adaptability* of the I-Score process, to enable adjustments to local considerations while maintaining the intervention's core components (i.e. I-Score completion by the patient via Opal prior to the clinic visit, review of scores by the physician in conjunction with the visit). Many peripheral components will be adaptable, such as the timing and number of reminders to complete the I-Score and how I-Score results are presented to providers on the ORMS dashboard.

Data collection

Quantitative component

The quantitative component will have three sources of data: 1) participant self-report; 2) electronic medical records; and 3) passive data (e.g., on app use to assess fidelity). At T1, T2 (3 months) and T3 (6 months), a study questionnaire will be administered to participants online or, if needed, with a hardcopy, especially to assess implementation outcomes.

At T1, the questionnaire will also ask about socio-demographics (e.g., year of birth, preferred language, sex, sexual orientation, ethnic group identity, immigration, education, income) and digital technology use as well as pose general health questions for patients (year of diagnosis with HIV, treatment satisfaction) and clinical practice questions for physicians (years practicing in HIV, current number of HIV patients). The measures of digital technology use are as follows: frequency of mobile device use (adapted from Schnall et al., 2018), having a health app on one's mobile device (Mahmood et al., 2019), extent of health app use (adapted from Balapour et al., 2019), confidence in reporting medical information using mobile technology (Balapour et al., 2019), and intention to report personal health data with a mobile device app, if asked by a provider (Balapour et al., 2019). All of this information will help contextualize the findings and describe the sample (see the Appendix for the patient and physician questionnaires). Clinical data, namely, HIV viral load in copies/mL, to determine viral suppression, will be extracted from patients' medical health record at the clinic, at T1 and T3.

Qualitative component

The qualitative component will have three sources of data: 1) 1-hour focus groups with all physicians (T2, T3); 2) 45-minute interviews with patients (T2, T3), until core theme saturation (an intermediate sample of 15 should be sufficient at each time point; Hennick et al., 2017); and 3) the Application Manager field notes, recorded on a standardized form (T1-T3). Focus groups and patient interviews will be conducted, if possible, through a teleconferencing platform such as Zoom. Participants will have the option of accessing the teleconference by telephone or the Internet. The patient's name will not be shown. Audio recordings of the focus groups and interviews will be manually transcribed, extracting nominal information. Each will be guided by a similar semi-structured interview schedule, in English or French, depending on preferred language. It will ask about the participants' experience with I-Score use and its implementation and about facilitating and impeding factors. The interview schedules can be found in the Appendix.

The schedule of study procedures for patients and physicians can be found in Table 1.

Study metrics and instruments

Details on the constructs assessed; the instruments and metrics used; the chosen thresholds for success, if applicable; the participant group contributing data; and the timing of data collection are presented in Table 2. Many of the chosen metrics are based on those recommended by Stover et al. (2020). Importantly, the authors emphasize the need to standardize evaluation metrics in patient-reported measure implementation and to distinguish between those used to assess perceptions of the innovation and those used to assess the implementation strategy. Not meeting the set thresholds for success, in this study, will signify that modifications are necessary before proceeding to a definitive trial (Thabane et al., 2010).

Table 1. Schedule of study procedures for participants

	**Timeline**			
**Procedure**	Prior to study start	Study start (baseline)	Month 3	Month 6
Be screened and/or consent	Patients<br>Physicians			
Attend educational meeting	Physicians			
Receive training on the I-Score measure and Opal	Patients<br>Physicians	Patients<br>Physicians	Patients<br>Physicians	Patients<br>Physicians
Complete the I-Score measure via Opal		Patients	Patients	Patients
Examine the I-Score measure results via the ORMS dashboard		Physicians	Physicians	Physicians
Attend HIV patient-physician visit (online or in person)		Patients<br>Physicians	Patients<br>Physicians	Patients<br>Physicians
Complete the post-visit checklist		Physicians	Physicians	Physicians
Complete the online sociodemographic questionnaire		Patients<br>Physicians		
Complete the online study questionnaire		Patients<br>Physicians	Patients<br>Physicians	Patients<br>Physicians
Possibly participate in an online qualitative interview			Patients	Patients
Participate in an online focus group			Physicians	Physicians
Receive compensation		Patients	Patients	Patients

Objective 1 -Evaluate perceptions of the I-Score intervention

Acceptability

The primary outcome of this pilot study will be acceptability, as measured by the Acceptability E-scale (AES) for web-based PROMs (Tariman et al., 2011). *Acceptability* is related to how agreeable, palatable, or satisfactory an intervention is perceived to be by stakeholders (Proctor et al., 2011). It will be measured, at T1, T2, and T3, with an adapted version of the AES for administration to both patients and physicians in the study. The scale has 6 items rated on a 5-point Likert scale that varies depending on the item. Example items of the original measure include "How would you rate your overall satisfaction with this computer program?", and "How easy was this computer program […] for you to use?" A summary score is obtained by adding the item scores (range: 6-30). A score of at least 24 (80% of maximum) indicates high acceptability and usability, as suggested by the scale developers.

Acceptability will also be measured at T1, T2 and T3 with a variant of the Net Promoters Score (NPS) used by England's National Health Service (NHS) and labelled the Friends and Family Test (NHS, 2014). The NPS is considered a measure of user satisfaction (Hamilton et al., 2014). A single question will be asked ("How likely are you to recommend the I-Score?") and rated on a 5-point Likert scale (1 = Extremely unlikely, 2 = Unlikely; 3 = Neither likely nor unlikely; 4 = Likely; 5 = Extremely likely). From this measure, the percentage recommending the I-Score will be calculated (score of 4 or 5), with a success threshold of 80% or more. An NPS-type score will also be calculated by creating three groups: promoters (score of 5), passives (score of 4), and detractors (score of 1-3). Subtracting the percentage of detractors from the promoters provides the NPS. NPS scores range from -100 to 100. A positive score (> 0) will be considered good (Stirling et al., 2019), and a score of ≥ 50, excellent.

Appropriateness

Appropriateness concerns the perceived fit or relevance of the intervention for the particular users, setting, or problem at hand (Proctor et al., 2011). It will be measured, at T1, T2, and T3, with two instruments. One concerns the perceived compatibility of the I-Score with the physicians' work. The perceived compatibility of an information technology innovation broadly relates to how consistent it is perceived to be with the potential users' values, needs, and past experiences (Moore and Benbasat, 1991). It will only be collected from providers, with a compatibility subscale developed by Moore and Benbasat (1991) which contains four items (e.g., "Using [the IT innovation] is compatible with all aspects of my work", "Using [the IT innovation] fits into my work style"). These are rated on a 7-point Likert scale, from Extremely disagree to Extremely agree, and averaged to produce the subscale score. A minimum average score of 5.5 is the threshold set for compatibility.

In addition, a 4-item scale, the Appropriateness of Intervention Measure (Weiner et al., 2017), will be completed by all participants. Example items include "This [evidence-based practice] seems fitting" and "This [evidence-based practice] seems like a good match." Items are scored on a five-point scale of agreement, from 1 = Completely disagree to 5 = Completely agree and averaged for a total score. An average score of at least 4 will indicate adequate appropriateness with this instrument.

Feasibility

Feasibility relates to the extent to which our I-Score intervention is successfully used or carried out within the study site (Proctor et al., 2011). To determine feasibility, data will be collected on the Consent rate, defined as the proportion of approached eligible patients and physicians who consent to participate. Individuals who choose not to participate will be asked to provide select sociodemographic information (sex, year of birth, preferred language) and their reason(s) with a checklist on a refusal form (see the Appendix). If 70% or more agree to participate, the study will be judged feasible on this aspect. We will also examine the Retention rate, indicated by the proportion of patients and physicians who complete the study. Eighty percent will be considered the benchmark for success. Missing I-Score data rates due to network failure as well as patient and provider non-completion of self-reported questionnaire data will be calculated as well. The criterion for success on this metric is at least 90%. Furthermore, participants will complete the Feasibility of Intervention Measure (Weiner et al.,

2017), at T1, T2 and T3, a four-item self-report measure that is appropriate for different stakeholder groups (e.g., patients, providers). Example items include "This [evidence-based practice] seems possible" and "This [evidence-based practice] seems doable." Average scores of at least 4, indicative of agreement on the 5-point response scale, will signify the I-Score intervention's feasibility.

Fidelity

Fidelity is the degree to which the intervention was implemented as specified in the protocol (Proctor et al., 2011). It will be indicated by patient and provider adherence to core components of the intervention. Thresholds for success, from T1 to T3, are: 90% patient completion of the I-Score prior to meeting with the physician; 90% provider review of the patient's I-Score results prior to or during the clinic visit.

Objective 2 -Evaluate the implementation strategy

Evaluation of the implementation strategy will be performed in relation to the same constructs as for the first objective. However, the assessment of acceptability, appropriateness, and fidelity will be solely based on analysis of qualitative data (see Table 2). As to feasibility, it will be assessed in terms of the rate of technical issues encountered and recorded in the Application Manager's notes, and the percentage of providers who participated in the implementation activities (i.e. education meeting, focus groups), with a success threshold set at 80% or more.

Objective 3 -Determine preliminary intervention and effectiveness outcome utility

This pilot study will collect data on one service outcome, patient management. It will be verified with a checklist submitted to participating physicians to allow them to record, per patient encounter, if they received the I-Score results on time, if they reviewed them prior to or during the clinic visit, if they were discussed during the visit, and if the I-Score identified concerning barriers. Then, they will check off any clinical actions that were taken based on the I-Score results or any related patient-provider discussion (e.g., recording issues in medical notes, referring to another health professional, ordering a test, changing a medication or treatment, providing advice or education). See the Appendix for the physician checklist.

The patient outcomes assessed in this pilot are barriers to adherence, adherence and viral load. Barriers to ART adherence will be assessed at T1, T2, and T3, with our previously described ePROM. Adherence will be examined with the Self-Rating Scale Item (SRSI) (Feldman et al., 2013) at T1, T2, and T3. It is a one item measure of treatment adherence (i.e. ''Rate your ability to take all your medications as prescribed'' [over the past 4 weeks], rated on a 6-point scale (i.e. Very poor, Poor, Fair, Good, Very good, and Excellent). Viral load, a clinical indicator of viral activity (e.g., infectiousness) and treatment response, will be treated as a dichotomous variable based on whether, as indicated in the patient's medical file, the HIV RNA viral load is detectable (over 50 copies), or not. Undetectability is a goal of HIV treatment. The most recent viral load test result at T1 and T3, will be collected.

Data analysis

Quantitative analysis

Time 1 questionnaires for people living with HIV and for HIV physicians will be summarized with descriptive statistics. For continuous variables, the minimum, the maximum, the mean, and the standard deviation will be reported. For ordinal and nominal qualitative variables, we will report absolute and relative frequencies (proportions).

As, specified, for most quantitative metrics relating to Objectives 1 and 2, as recommended for pilot studies, score targets were set to evaluate the ability to proceed to a definitive trial (Thabane et al., 2010). For people living with HIV and HIV physicians, continuous outcomes expressed on a Likert scale will be summarized with the minimum, the maximum, the mean, and the standard deviation at T1, T2 and T3. Binary outcomes (yes or no) will be reported with absolute and relative frequencies (proportions) at T1, T2 and T3. The means and proportions of T1, T2 and T3 will be confronted with their corresponding thresholds for success, presented in Table 2. To study the tendency of means and proportions for people living with HIV over time, a Linear Mixed Model (LMM) or a

Generalized Linear Mixed Model (GLMM) will be used, for continuous and binary outcomes, respectively. The response variable of each model will be the corresponding outcome and the independent variable will be the time (T1, T2 and T3). The null hypothesis of no time effect on the corresponding outcome will be tested with a Student's t-test on the regression coefficient. If the null hypothesis is rejected, we will perform post-hoc Student's paired t-tests between all combinations of time points to show between which time points means and proportions differed significantly. Additionally, to verify if each threshold for success is met at the end of the study, we will test the null hypothesis that each mean or proportion at T3 is inferior to its corresponding threshold with a Student's t-test. For all analyses, a significance level of 5% will be adopted. Finally, where appropriate, Cronbach's alpha will be calculated to evaluate the internal consistency of subscales.

Regarding the patient outcomes of Objective 3, barriers to adherence and adherence will be summarized with the minimum, the maximum, the mean, and the standard deviation. Viral load will be reported by absolute and relative frequencies, as it is considered a dichotomous variable. For the service outcome obtained from the physician checklist, we will report the proportion of clinic visits when physicians took action based on the I-Score results, among the visits where an adherence barrier of concern was identified by physicians. Proportions will be reported for T1, T2 and T3 and globally, across time periods. To evaluate evidence of a statistically significant difference in our chosen effectiveness outcomes, we will run a Student's paired t-test for barriers to adherence and adherence and a McNemar test for viral load, between T1 and T3. To complete the analysis of the service outcome, we will use a logistic regression model, considering only the visits where an adherence barrier of concern was identified by physicians. The dependent variable is the binary variable of whether or not an action was taken by physicians and the independent variable is the time, considered as a factor with three independent levels (T1, T2 and T3). We will test the null hypothesis that time has no effect on the probability of taking action, with a t-test on the regression coefficient. We will conclude the analysis by testing the null hypothesis of equality of proportions between all pairwise combinations of time points, with a Student's t-test between two proportions, performing a Bonferroni correction for multiple tests. For all analyses, a significance level of 5% will be adopted.

Qualitative analysis

The study's qualitative material (i.e. focus groups, interviews, Application Manager notes) will be submitted to content analysis (Elo & Kyngäs, 2008), focusing on the manifest content. Deductive content analysis will be favored, allowing implementation barriers and facilitators identified to be categorized with an existing framework, while remaining open to emerging categories. Deductive content analysis allows categories to be compared at different periods, fitting with the study's longitudinal design (Vaismoradi et al., 2014). For this purpose, the Consolidated Framework for Implementation Research (CFIR) will be used (Damschroder et al., 2009). Analysis will involve three phases (Elo & Kyngäs, 2008): 1) preparation, when the analyst attempts to get a sense of the entire dataset through immersion in the data; 2) organizing, during which an unconstrained categorization matrix will be devised with the CFIR's constructs, and the data will be coded, accordingly. At this point, the qualitative data management software, Atlas.ti version 8, will be used to code and categorize the material; and 3) reporting, which involves presenting the described contents (meanings) of the categories and addressing trustworthiness (Elo & Kyngäs, 2008). A product of these analyses will be matrices of facilitators, barriers and potential solutions raised by patients and physicians, at each main qualitative data collection period, using the CFIR. These will allow for the tracking of categories over time (Damschroder et al., 2015), to help identify patterns.

For the cyclical small tests of change of the implementation strategy, consistent with the approach by Keith et al. (2017), the qualitative data will be coded and categorized with the CFIR. We will further structure and document our cyclical small tests of change by drawing on the iterative Plan-Do-Study-Act (PDSA) approach for quality improvement (Taylor et al., 2014). During the 'plan' stage, the stakeholder feedback collected will help to periodically identify and document factors that are affecting the intervention and associated changes to the implementation and/or peripheral components of the intervention that could lead to improvement. Related predictions will be explicitly articulated (Taylor et al., 2014). During the 'do' stage, changes will be tested. At the 'study' stage, the successfulness of the change will be evaluated against the prediction(s) and documented with subsequent qualitative or quantitative data, per the study's design, and the Application Manager's field notes. The

‘act’ stage will see further adaptations, depending on the successfulness of the change, and/or the initiation of another cycle of change. For each PDSA cycle undertaken, all decisions and relevant information will be recorded, following the PDSA theoretical framework developed by Taylor et al., (2014).

Mixed methods analysis

The quantitative and qualitative data will be analyzed separately and subsequently brought together for comparison, for a more complete interpretation of the results. Areas of convergence and divergence will be highlighted.

Participant security

Adverse events (AE’s)

An adverse event (AE) is any untoward medical occurrence in a patient-participant who is administered an intervention/treatment which does not necessarily have a causal relationship with this intervention/treatment. AE’s are therefore temporally related to participation in the study.

Participants in this study, whether they are involved in the interview or in filling out the study questionnaires, face no direct physical risk of participating as they will not be submitted to any pharmaceutical or invasive medical interventions. If an AE occurs in a patient who participated in this study, it is probable that it will involve a negative emotional reaction (e.g., distress, anxiety) caused by the qualitative interview about the intervention or by completing the I-Score PROM (e.g., on difficulties adhering to ART). Participants will be informed that they can be referred to a mental health professional within a reasonable amount of time should they experience significant emotional distress as a result of their participation in this study.

Documentation of AE’s

Any AE that occurs from the moment a participant signs the consent form to the time they leave the study will be documented by the study’s research personnel in the appropriate Case Report Form. The dates of the beginning and end of the AE, its severity (Grade), and the principal investigator’s judgment about the relationship of the AE to participation in the study (e.g., “definitely related” to “not related”) will be recorded on this form.

Expected study contributions

ePROM use for clinical practice with individuals living with HIV is limited. To our knowledge, this is the first pilot study of an intervention aimed at implementing systematic consideration of patient-identified ART adherence barriers with an ePROM in routine HIV care. This pilot study will help build needed knowledge on impediments to and strategies for implementing ePROMs in HIV care (Kall et al., 2020). As such, it acts as a standalone study, providing useful and rich data to others considering similar interventions in similar contexts. It will generate data that will improve understanding of conditions for successful implementation as well as test and solidify the implementation strategy. Furthermore, it may shed light on the mechanisms of ePROM interventions. Overall, it will produce useful data to design a definitive effectiveness trial of the I-Score intervention.

Conducted within a CIHR SPOR Mentorship Chair research program, awarded to the PI, Dr. Lebouché, it will help train HIV researchers in patient-oriented research. The PROM initiative concerned by this study also challenges traditional care paradigms with a more patient-centered approach. It promises to shift an HIV treatment paradigm emphasizing biomedical markers (i.e. viral load) in adherence management. Systematic monitoring of patient-reported adherence barriers will allow for a more preventative approach and help ensure adherence management addresses patients’ priorities. The I-Score PROM includes only the most highly valued barriers in terms of relevance and importance to HIV care, as rated by people living with HIV in our Delphi consultation (Engler et al., 2019). As to the app through which the PROM is administered and its features, it may help redress the patient-provider knowledge imbalance and empower patients in their care (Rigby et al., 2015).

Scientific contributions

Communication of results and knowledge transfer

Scientific

The results derived from this study will be presented at national (e.g., Canadian Association for HIV Research) and international (e.g., Conference on Retroviruses and Opportunistic Infections; IAS Conference on HIV Pathogenesis, Treatment and Prevention) scientific conferences. We expect to publish up to four scientific articles in peer-reviewed journals included in common medical databases (e.g., PubMed, Medline). These will likely include: 1) a publication of the study's research protocol; 2) a publication of the quantitative data; 3) a publication of the qualitative data; 4) a publication of the mixed methods analysis. The targeted periodicals will be selected based on the nature of the manuscript and their potential to reach the desired audience.

Clinical

Presentations based on the project and its results will also be given to healthcare professionals working in HIV and its treatment (e.g., Chronic Viral Illness Service, Clinique Médicale L'Actuel, etc).

Community

We will present the results of the project at conferences that attract both HIV/AIDS associations and clinicians, particularly that of the *société française de lutte contre le sida* in France and that of the Canadian Association for HIV Research in Canada (CAHR/ACRV).

Publication policy

The status of author for any publication produced in the course of this study will be attributed according to criteria of the « International Committee of Medical Journal Editors » (ICMJE). To be included as a co-author, the individual must have made a substantial contribution to these areas: 1) study conception, design or data collection, analysis or interpretation; 2) drafting the article or revising its main intellectual content; and 3) final approval of the version submitted for publication. The author must meet all three conditions, inclusively. Contributors who do not meet the criteria will be mentioned in the acknowledgements, where their particular contribution will be described.

Any source of funding for the study will be detailed in the area specified by the publication (e.g., conflicts of interests, acknowledgements) and in accordance with the funding body's requirements.

Project timeline

The project timeline is presented in Figure 3. Broad milestones are as follows:

- January-February 2021: Physician and patient enrollment
- March 2021-August 2021: Data collection
- April 2021-October 2021: Data analysis
- September 2020-December 2021: Presentation and manuscript preparation (beginning with the protocol manuscript)

Anticipated problems

There are many potential barriers to implementing PROMs in care, such as provider reticence (e.g., due to concerns for increased workload). Our multi-pronged implementation strategy directly seeks to mitigate numerous common barriers to implementing PROMs in care (Foster et al., 2018). For details, see Table 1.

Currently, physicians have been advised to use telemedicine and teleconsultations, whenever possible, to limit the spread of the disease (American Medical Association, 2020, Royal College of Physicians and Surgeons of Canada, 2020). Given the uncertain evolution of the COVID-19 pandemic and associated public health response, methodological adjustments to this study may be required to further limit in-person participant visits with physicians and research team members. The REB will be informed if this is the case.

Project management

Scientific committee

The pilot study team is multidisciplinary and has expertise in HIV clinical practice, biostatistics, trial design, health informatics, qualitative methods, psychometrics, epidemiology, PROM use for chronic disease management, implementation science, and HIV community concerns around treatment and adherence. The PI also has expertise in stakeholder engagement (e.g., Lessard et al., 2019), having notably been awarded a CIHR Mentorship Chair in patient-oriented research. With this Chair, all affiliated studies, prior to initiation, are submitted to a stakeholder committee with patient members for feedback (e.g., on study relevance, participation burden, data collection tools). These resources and competencies as well as a history of past collaboration between most team members will ensure the successful conduct and important contribution of this study. CTN funding for the pilot will allow us to secure matching funding for trainees through the MITACS Accelerate program, further ensuring the pilot's advancement.

Research personnel

The research personnel is decidedly multidisciplinary as well, with a wide range of relevant expertise.

David Lessard, PhD, is an anthropologist and a research assistant specialized in patient engagement. He will coordinate and conduct consultations with the I-Score Consulting Team. He will also participate in qualitative data collection.

Adriana Rodriguez has a PhD in biomedical sciences (immunology) and has experience coordinating clinical studies. Her role in this study will be to coordinate this study and manage ethics submissions.

Yuanchao Ma, M.Sc.A, is a mechanical engineer and will act as the study's Application Manager, to ensure participant training and data quality.

Serge Vicente, PhD(c), is a statistician and will help plan and conduct the statistical analyses of this study.

Collaborators

The I-Score Consulting Team has contributed patient expertise to the I-Score Study (CTN 283) since 2015. This team has participated in study decision-making, knowledge translation, and directly as participants in -Score related research (Lessard et al., 2019). It is a diverse yet cohesive and experienced committee with a range of professional skills and interests.

Patrick Keeler has been working with us for years, providing a community perspective on research and helping directly with our research program (e.g., recruitment). Originally, the Coordinator of Treatment Information and affiliated with the Aging and HIV Program at AIDS Community Care Montreal, he is now with the 'Cercle Orange', a cost-free referral and support service for people living with HIV in Montreal without access to health care, based at la COCQ-sida. He brings significant expertise on HIV community needs.

Ethical considerations

Confidentiality

All the information collected about participants during the study will remain confidential as the law demands. To protect participant privacy, their information will be identified with an ID code of numbers and/or letters. Only the investigator in charge of the study will know the numbers and/or letters that link them to participants. Any study paper documents containing nominal information on the participants will be stored under lock and key and they will only be accessible to the project investigators and personnel. The questionnaires and audio recordings of the interviews will be identified by an alphanumeric code, assigned to each participant. All electronic files (e.g., interview transcriptions) will also be identified by a code and will require a password to access them. The computers used for analysis or record keeping will be protected by a password.

When the study is over, all study documents and data will be kept in a secure place at the Chronic Viral Illness Service of the McGill University Health Centre for 2 years. Afterwards, they will be archived at Iron Mountain in Laval, Quebec. Once the study's results have been published, it will be kept there for 7 years.

There are concerns for the collection of data on teleconferencing platforms such as Zoom for research with human participants. Care will therefore be taken to ensure confidentiality when configuring and recording the teleconference meeting. For example, teleconferences will require a password, and, when recording, "Local recording" will be 'on,' "Hosts can give participants the permission to record locally" will be 'off,' and "Cloud recording" will be 'off.'

Obtaining consent

Both patients and physicians will receive an informed consent form to read (by email or in hand), to supplement the explanations provided by the research coordinator or study staff. To maintain distancing during the COVID-19 pandemic, remote consent will be favored. Both physicians and patients will have the option of printing, signing, and electronically returning the consent form (whether by taking a photo of or scanning the signed form). As physicians and some patients will be physically present at the CVIS for in-person medical appointments, they will be able to receive and sign a hardcopy of the ICF on site, prior to participation. For patients without a printer and not visiting the clinic prior to participation, oral consent will be possible, as this is a low-risk study for participants. This will involve having a third-party present when obtaining consent during a scheduled teleconference or phone conversation. Both the impartial witness and the person obtaining consent will sign the ICF. The ICF will be later signed by the participant at their next regular visit to the clinic (T0).

Ethics approval

For the Chronic Viral Illness Service site (Royal Victoria Hospital), prior to implementing the study, the study documents, including the research protocol, consent forms and the data collection tools will be submitted for approval by an appropriate ethics and scientific committee of the Research Institute of the McGill University Health Centre.

Good clinical practices statement

This study will be conducted in accordance with the applicable Health Canada regulations, International Conference on Harmonisation (ICH) guidelines on current Good Clinical Practice (GCP), and the Declaration of Helsinki.

Protocol deviations and violations

Any requested minor exemptions may be considered on a case by case basis and documented. Deviations or violations of the protocol will be reported to the project manager.

Source documents

Some quantitative data will be collected with Case Report Forms (CRF) developed specifically for this study. This will include data derived from patients' medical files (clinical data). Source documents include any recordings of observations or notations and all reports and records necessary for the evaluation and reconstruction of the study. Source documents should support the data collected with the CRF's.

All of the study data collected may be reviewed by staff of the McGill University Health Centre's (MUHC) Research Ethics Board to ensure the quality, integrity and safety of this study.

Relevant skills of the scientific committee members

Bertrand, Lebouché, MD, PhD	Dr. Lebouché (Associate Professor, McGill University) is an HIV physician with expertise in stakeholder engagement, mentorship, and research ethics. He will ensure the overall supervision of the study and

	staff/trainees.
Kim Engler, PhD	Dr. Engler (Research Associate, Research Institute, MUHC) has expertise in qualitative methods and will lead portions of data analysis and manuscript/presentation development. She led content development and validation of the I-Score patient-reported outcome measure.
Tibor Schuster, PhD	Dr. Schuster (Associate Professor, McGill University) is a biostatistician with extensive expertise applying quantitative methods in a broad range of diagnostic, clinical therapeutic and epidemiological studies. He will plan and supervise the statistical analyses.
John Kildea, PhD	Dr. Kildea (Assistant Professor, McGill University) is a medical physicist and co-developer of the Opal patient portal/smartphone app. He will be involved in the app's implementation at the CVIS and help supervise Yuanchao Ma.
Tarek Hijal, MD	Dr. Hijal (Associate Professor, McGill University) is a physician specialized in radiation oncology. He is a co-developer of the Opal patient portal/app.
Joseph Cox, MD	Dr. Cox (Associate Professor, McGill University) is an HIV physician and will contribute expertise in implementation science.
Sara Ahmed, PhD	Dr. Ahmed (Associate Professor, McGill University) will provide expertise in the use of patient-reported outcome measures for the management of chronic disease and computer-enabled self-management interventions.
Nitika Pant Pai, MD	Dr. Pai (Assistant Professor, McGill University) is a physician and epidemiologist who will provide renowned expertise in app-based health technology implementation.
Marina Klein, MD	Dr. Klein (Professor, McGill University) is an HIV physician and epidemiologist, with extensive clinical and research experience.
Alexandra de Pokomandy, MD	Dr. de Pokomandy (Associate Professor, McGill University) is an HIV physician and epidemiologist who will especially bring expertise in HIV-positive women's health to the study.
Sofiane Achiche, PhD	Dr. Achiche (Professor, Polytechnique de Montreal) is a mechanical engineer with expertise in artificial intelligence and machine learning. He will provide input on how Opal and the I-Score may be adapted to problems encountered during implementation.

Budget

**Project Component**	**Amount**
Staff (includes 19% fringe benefits as per RI-MUHC policies)	
1. Application manager ($30/hr, 10 hrs x 32 weeks)	$9,600
2. Research assistant ($30/hr, 10 hrs x 32 weeks)	$9,600
3. Research coordinator ($30/hr, 5 hours x 32 weeks)	$4,800
4. Transcriber ($25/hr, 7 hours x 30 interviews = 5,250) + ($25, 9 hours x 2 focus groups = 450)	$5,700
5. Qualitative data analyst/manuscript prep ($30/hr, 7 hrs x 40 weeks)	$8,400
6. Quantitative data analyst/manuscript prep ($30/hr, 7 hrs x 40 weeks)	$8,400
Supplies and services	

1.	Patient compensation for the interviews ($30 x 15 patients x 2 times)	$900
2.	Patient compensation for questionnaire completion ($20 x 30 patients x 3 times)	$1800
**Total**		**$49,200**

References

American Medical Association. AMA quick guide to telemedicine in practice. Updated June 22, 2020. Access July 3, 2020. https://www.ama-assn.org/practice-management/digital/ama-quick-guidetelemedicine-Practice

Balapour A, Reychav I, Sabherwal R, Azuri J. Mobile technology identity and self-efficacy: implications for the adoption of clinically supported mobile health apps. *International journal of information management* 2019;49:58-68. doi:10.1016/j.ijinfomgt.2019.03.005

Barfod TS, Hecht FM, Rubow C, et al. Physicians' communication with patients about adherence to HIV medication in San Francisco and Copenhagen: a qualitative study using Grounded Theory. *BMC Health Serv Res* 2006;6:154.

Beach MC, Roter DL, Saha S, et al. Impact of a brief patient and provider intervention to improve the quality of communication about medication adherence among HIV patients. *Patient Educ Couns* 2015;98(9):1078-1083.

Bezabhe WM, Chalmers L, Bereznicki LR, Peterson GM. Adherence to antiretroviral therapy and virologic failure: a meta-analysis. *Medicine* (Baltimore). 2016;95(15):e3361.

Boyce M, Browne J. Does providing feedback on patient-reported outcomes to healthcare professionals result in better outcomes for patients? A systematic review. Quality of Life Research. 2013;22(9):2265–2278.

Cohen, J., Beaubrun, A., Bashyal, R. *et al.* Real-world adherence and persistence for newly-prescribed HIV treatment: single versus multiple tablet regimen comparison among US medicaid beneficiaries. *AIDS Res Ther* 2020;17:12. https://doi.org/10.1186/s12981-020-00268-1

Creswell JW, Plano-Clark VL. Designing and Conducting Mixed Methods Research. 2nd Edition. London, UK: Sage Publications 2011.

Curran GM, Bauer M, Mittman B, et al. Effectiveness-implementation hybrid designs: combining elements of clinical effectiveness and implementation research to enhance public health impact. *Med Care* 2012;50(3):217–226.

Damschroder LJ, Aron DC, Keith RE, Kirsh SR, Alexander JA, Lowery JC. Fostering implementation of health services research findings into practice: a consolidated framework for advancing implementation science. *Implementation science* 2009;4(1). doi:10.1186/1748-5908-4-50

Department of Health and Human Services (DHHS). Panel on Antiretroviral Guidelines for Adults and Adolescents. Guidelines for the Use of Antiretroviral Agents in Adults and Adolescents Living with HIV. http://www.aidsinfo.nih.gov/ContentFiles/ AdultandAdolescentGL.pdf. (accessed April 7, 2020).

Elo S, Kyngäs H. Original methodology: the qualitative content analysis process. *J Adv Nurs* 2008;62(1):107-115.

Engler K, Ahmed S, Lessard D, Vicente S, Lebouché B. Assessing the content validity of a new patient-reported measure of barriers to antiretroviral therapy adherence for electronic administration in routine HIV care: proposal for a web-based Delphi study. *JMIR Res Protoc* 2019;8(8):e12836.

Engler K, Lènàrt A, Lessard D, Toupin I, Lebouché B. Barriers to antiretroviral therapy adherence in developed countries: a qualitative synthesis to develop a conceptual framework for a new patient-reported outcome measure. *Aids care* 2018;30(Sup1):17-28. doi:10.1080/09540121.2018.1469725

Feldman BJ, Fredericksen RJ, Crane PK, et al. Evaluation of the single-item self-rating adherence scale for use in routine clinical care of people living with HIV. *AIDS Behav* 2013;17(1):307-318.

Foster A, Croot L, Brazier J, et al. The facilitators and barriers to implementing patient reported outcome measures in organisations delivering health related services: a systematic review of reviews. *J Patient Rep Outcomes* 2018;2:46.

Fredericksen R, Crane PK, Tufano J, et al. Integrating a web-based, patient-administered assessment into primary care for HIV-infected adults. *J AIDS HIV Res* 2012;4(2):47–55.

Genberg BL, Lee Y, Rogers WH, et al. Four types of barriers to adherence of antiretroviral therapy are associated with decreased adherence over time. *AIDS Behav* 2015;19(1):85-92.

Greenhalgh J, Dalkin S, Gooding K, et al. Functionality and feedback: a realist synthesis of the collation, interpretation and utilisation of patient-reported outcome measures data to improve patient care. *Health Serv Deliv Res* 2017;5(2).

Greenhalgh J, Long AF, Flynn R. The use of patient reported outcome measures in routine clinical practice: lack of impact or lack of theory? *Social Sci Med* 2005;60:833e43.

Hennink MM, Kaiser BN, Marconi VC. Code saturation versus meaning saturation: how many interviews are enough? *Qualitative health research* 2017;27(4):591-608. doi:10.1177/1049732316665344

International Society for Quality of Life Research [ISOQOL] (prepared by Aaronson N, Choucair A, Elliott T, Greenhalgh J, Halyard M, Hess R, Miller D, Reeve B, Santana M, Snyder C). User's Guide to Implementing Patient-Reported Outcomes Assessment in Clinical Practice, Version: November 11, 2011.

Ishaque S, Karnon J, Chen G, Nair R, Salter AB. A systematic review of randomised controlled trials evaluating the use of patient-reported outcome measures (proms). *Quality of life research* 2019;28(3):567-592. doi:10.1007/s11136-018-2016-z

ISO 9241-11. Ergonomics of human-system interaction — part 11: usability: definitions and concepts. International Organization for Standardization, 2018.

Kall M, Marcellin F, Harding R, Lazarus JV, Carrieri P. Patient-reported outcomes to enhance person-centred HIV care. *The lancet hiv* 2020;7(1):68. doi:10.1016/S2352-3018(19)30345-5

Keith RE, Crosson JC, O'Malley AS, et al. Using the Consolidated Framework for Implementation Research (CFIR) to produce actionable findings: a rapid-cycle evaluation approach to improving implementation. *Implement Sci* 2017;12:15.

Kildea J, Battista J, Cabral B, et al. Design and development of a person-centered patient portal using participatory stakeholder co-design. *Journal of medical internet research* 2019;21(2):11371. doi:10.2196/11371

Kjær ASHK, Rasmussen TA, Hjollund NH, Rodkjaer LO, Storgaard M. Patient-reported outcomes in daily clinical practise in hiv outpatient care. *International journal of infectious diseases* 2018;69:108-114. doi:10.1016/j.ijid.2018.02.015

Laws MB, Beach MC, Lee Y, et al. Provider-patient adherence dialogue in HIV care: results of a multisite study. *AIDS Behav* 2013;17(1):148–159.

Lessard D, Engler K, Toupin I, Routy J-P, Lebouché B. Evaluation of a project to engage patients in the development of a patient-reported measure for hiv care (the i-score study). *Health expectations* 2019;22(2):209-225. doi:10.1111/hex.12845

Mahmood A, Kedia S, Wyant DK, Ahn S, Bhuyan SS. Use of mobile health applications for health-promoting behavior among individuals with chronic medical conditions. *Digit Health* 2019;5:2055207619882181. doi:10.1177/2055207619882181

Malta M, Petersen ML, Clair S, Freitas F, Bastos FI. Adherence to antiretroviral therapy: a qualitative study with physicians from Rio de Janeiro, Brazil. *Cad Saúde Pública* 2005, 21(5):1424-1432.

Marandino L, Necchi A, Aglietta M, Di Maio M. COVID-19 emergency and the need to speed up the adoption of electronic patient-reported outcomes in cancer clinical practice. *JCO Oncol Pract*. 2020;16(6):295-298. doi:10.1200/OP.20.00237

Mercieca-Bebber R, Palmer MJ, Brundage M, et al. Design, implementation and reporting strategies to reduce the instance and impact of missing patient-reported outcome (PRO) data: a systematic review. BMJ Open 2016;6:e010938. doi:10.1136/bmjopen-2015- 010938

Miller LG, Liu H, Hays RD, et al. How well do clinicians estimate patients' adherence to combination antiretroviral therapy?. *J Gen Intern Med* 2002;17(1):1-11.

Ministère de la santé et des services sociaux. La thérapie antirétrovirale pour les adultes infectés par le VIH - Guide pour les professionnels de la santé du Québec. Gouvernement du Québec. Last updated February 2, 2019. Retrieved August 7, 2020 from: https://publications.msss.gouv.qc.ca/msss/document-000733/?&date=DESC&sujet=vih-sida&critere=sujet

Moore, G., & Benbasat, I. (1991). Development of an instrument to measure the perceptions of adopting an information technology innovation. *Information Systems Research, 2*(3), 192-222. doi:10.1287/isre.2.3.192

Murray E, Hekler EB, Andersson G, et al. Evaluating digital health interventions: key questions and approaches. *Am J Prev Med* 2016;51(5):843-851.

National Health Service England. NHS England Review of the Friends and Family Test. 2014. Retrieved August 13, 2020 from https://www.england.nhs.uk/wp-content/uploads/2014/07/fft-rev1.pdf

O'Cathain A, Murphy E, Nicholl J. The quality of mixed methods studies in health services research. *J Health Serv Res Policy* 2008;13(2):92-98. doi:10.1258/jhsrp.2007.007074

Ortego C, Huedo-Medina TB, Llorca J, et al. Adherence to highly active antiretroviral therapy (haart): a meta-analysis. *AIDS Behav* 2011;15(7):1381-1396.

Peters DH, Tran NT, Adam T. Implementation research in health: a practical guide. Alliance for Health Policy and Systems Research. World Health Organization 2013.

Pinnock H, Barwick M, Carpenter CR, Eldridge S, Grandes G, Griffiths CJ et al. Standards for reporting implementation studies (StaRI) statement. BMJ 2017;356:i6795.

Powell BJ, McMillen JC, Proctor EK, et al. A compilation of strategies for implementing clinical innovations in health and mental health. *Med Care Res Rev* 2012;69(2):123-157.

Powell BJ, Waltz TJ, Chinman MJ, et al. A refined compilation of implementation strategies: results from the expert recommendations for implementing change (eric) project. *Implement Sci* 2015;10:21-21.

Proctor E, Silmere H, Raghavan R, et al. Outcomes for implementation research: conceptual distinctions, measurement challenges, and research agenda. *Adm Policy Ment Health* 2011;38(2):65-76.

Public Health Agency of Canada. *Summary: Estimates of HIV Incidence, Prevalence and Canada's Progress on Meeting the 90-90-90 HIV targets, 2016*. Public Health Agency of Canada, 2018. Retrieved August 7, 2020 from: https://www.canada.ca/en/public-health/services/publications/diseases-conditions/summary-estimates-hiv-incidence-prevalence-canadas-progress-90-90-90.html

Rigby M, Georgiou A, Hyppönen H, et al. Patient portals as a means of information and communication technology support to patient-centric care coordination – the missing evidence and the challenges of evaluation. *Yearbook of medical informatics*. 2015;24(01):148-159.

Royal College of Physicians and Surgeons of Canada. Telemedicine and virtual care guidelines (and other clinical resources for COVID-19). Updated May 21, 2020. Accessed July 3, 2020. http://www.royalcollege.ca/rcsite/documents/about/covid-19-resources-telemedicine-virtual-care-e#qc

Rutherford C, Campbell R, King M, et al. Implementing patient-reported outcome measures into clinical practice across nsw: mixed methods evaluation of the first year. *Applied research in quality of life* 2020. doi:10.1007/s11482-020-09817-2

Santana MJ, Feeny D. Framework to assess the effects of using patient-reported outcome measures in chronic care management. *Qual Life Res* 2014;23(5):1505-13.

Schnall, R., Cho, H., & Liu, J. (2018). Health information technology usability evaluation scale (health-itues) for usability assessment of mobile health technology: Validation study. *Jmir Mhealth and Uhealth 6*(1), 4. doi:10.2196/mhealth.8851

Shubber Z, Mills EJ, Nachega JB, et al. Patient-reported barriers to adherence to antiretroviral therapy: A systematic review and meta-analysis. *PLoS Med* 2016;13(11):e1002183.

Sim J, Lewis M. The size of a pilot study for a clinical trial should be calculated in relation to considerations of precision and efficiency. *Journal of Clinical Epidemiology* 2011:65;301-308.

Stover, A.M., Haverman, L., van Oers, H.A. *et al.* Using an implementation science approach to implement and evaluate patient-reported outcome measures (PROM) initiatives in routine care settings. *Qual Life Res* 2020. https://doi.org/10.1007/s11136-020-02564-9

Tariman JD, Berry DL, Halpenny B, Wolpin S, Schepp K. Validation and testing of the Acceptability E-scale for web-based patient-reported outcomes in cancer care. *Appl Nurs Res* 2011;24(1):53-58. doi:10.1016/j.apnr.2009.04.003

Taylor MJ, McNicholas C, Nicolay C, *et al*. Systematic review of the application of the plan–do–study–act method to improve quality in healthcare. *BMJ Qual Saf 2014;23:290-298.*

Thabane L, Ma J, Chu R, et al. A tutorial on pilot studies: the what, why and how. *BMC Med Res Methodol* 2010;10:1.

Toupin I, Engler K, Lessard D, et al. Developing a patient-reported outcome measure for hiv care on perceived barriers to antiretroviral adherence: assessing the needs of hiv clinicians through qualitative analysis. *Quality of life research* 2018;27(2):379-388. doi:10.1007/s11136-017-1711-5

Vaismoradi M, Turunen H, Bondas T. Content analysis and thematic analysis: implications for conducting a qualitative descriptive study. *Nursing and Health Sciences* 2014;15(3):398-405.

Valderas JM, Kotzeva A, Espallargues M, Guyatt G, Ferrans CE, Halyard MY, Revicki DA, Symonds T, Parada A, Alonso J. The impact of measuring patient-reported outcomes in clinical practice: a systematic review of the literature. Quality of Life Research. 2008;17(2):179–193.

Weiner BJ, Lewis CC, Stanick C, et al. Psychometric assessment of three newly developed implementation outcome measures. *Implement Sci* 2017;12(1):1-12.

Wilson IB, Laws MB, Safren SA, et al. Provider-focused intervention increases adherence-related dialogue but does not improve antiretroviral therapy adherence in persons with HIV. *J Acquir Immune Defic Syndr* 2010;53(3):338–347.

Table 2. Relationship between the identified facilitators/barriers of PROM implementation, the implementation framework (CFIR), and the pilot study's implementation strategies.

Stage of PROM implementa-tion*	Facilitator/ barrier*	CFIR framework construct**	Addressed	
			Prior to the pilot	Within the pilot: Implementation strategy***
**Purpose**: Motivations for implementing and objectives of using PROM	The perceived reason for implementing a PROM is important -if external, impact can be mixed	Intervention characteristics: Intervention source<br><br>Outer setting: External policy (e.g. guidelines)	Both individuals with HIV and providers in Canada and France were involved throughout the development of the PROM, including in needs assessments and content validation.	Educate: Conduct educational meetings<br><br>*An educational meeting will cover the origins of the PROM and how it was shaped through stakeholder engagement. The PROM intervention will also be presented as consistent with HIV treatment guidelines on the monitoring of adherence barriers.*
	The PROM should be to support the management of patients, rather than solely used as an audit tool	Inner setting: Implementation climate-Compatibility	The measure's main purpose is to aid in patient management and secondarily, it will serve as a research tool. It is not designed for use in performance management.	Educate: Conduct educational meetings<br><br>*The educational meeting will emphasize the patient management function of the PROM, clearly establishing that results are not used to monitor clinician performance, despite the collection of a service outcome in the pilot.*
**Designing**: Deciding the PROM process and how to implement it	Involve clinicians and patients in designing the PROM process	Intervention characteristics: Intervention source<br><br>Intervention characteristics: Design quality	Both individuals with HIV and providers in Canada and France were involved throughout the development of the PROM, including in needs assessments and content validation.<br><br>This pilot study is conducted in part so that the PROM process can be adapted to local patient and physician concerns.	Plan: Build buy-in -Involve patients/ consumers<br><br>Plan: Organize clinician implementation team meetings<br><br>*Participants will have several opportunities to provide feedback on the intervention and its implementation. Physicians will participate in focus groups (T2, T3) and patients will participate in individual interviews (T2, T3).*
	Consider the needs and resources of clinicians and patients when designing the PROM process	External setting: Patient needs and resources<br><br>Inner setting: Implementation climate-Compatibility	Both individuals with HIV and providers in Canada and France were involved throughout the development of the PROM, including in needs assessments and in content validation with Delphi techniques. Patients and providers were also involved in designing the smartphone application to administer the PROM.	Plan: Build buy-in -Involve patients/ consumers<br><br>Plan: Organize clinician implementation team meetings<br><br>Educate: Conduct ongoing training<br><br>Educate: Provide ongoing consultation<br><br>*These educational strategies will be enacted through the hiring of a Application Manager who will*

				train and assist patients and providers in the PROM process. Patients will be individually trained.
	The PROM process should limit complexity and have compatibility with current ways of working and clinicians' values	Intervention characteristics: Complexity<br><br>Inner setting: Implementation climate-Compatibility	See above response.<br><br>In addition, PROM completion and results examination was scheduled to coincide with regular clinic appointment times, to limit disruption.<br><br>The use of a smartphone app was also chosen to simplify the PROM process.	Plan: Build buy-in -Involve patients/ consumers<br><br>Plan: Organize clinician implementation team meetings<br><br>Educate: Conduct ongoing training<br><br>Educate: Provide ongoing consultation
	Have adaptability within the PROM process	Intervention characteristics: Adaptability	-	Promote adaptability<br><br>*We have distinguished between the core components of the intervention and those aspects which can be tailored to local needs.*<br><br>Quality management: Conduct cyclical small tests of change<br><br>*Building on feedback received through participating patient and physician feedback and the Application Manager's field notes, we will use a rapid-cycle evaluation approach, using the CFIR, to improve implementation.*
	Choose PROMs which are perceived as relevant and appropriate	Intervention characteristics: Evidence strength and quality<br><br>Characteristics of individuals: Knowledge and beliefs about the intervention	The Delphi which consulted individuals with HIV and providers on the actionability, and relevance of the PROM's content served in the final selection of the PROM's items.	Educate: Conduct educational meetings<br><br>*The educational meeting will provide data on the perceived importance and relevance of the PROM, including the results of the Delphi.*
**Preparing**: Getting an organization and its staff ready to use it	Convince clinicians about the validity, reliability and utility of PROMs	Characteristics of individuals: Knowledge and beliefs about the intervention	The PROM underwent an intensive validation process with patient and provider engagement.	Educate: Conduct educational meetings<br><br>*The educational meeting will provide data on the psychometric properties of the PROM.*
	Engage clinicians in the process	Process: Engaging (e.g. education, training)	-	Plan: Organize clinician implementation team meetings<br><br>*See all strategies aimed to educate.*
	Provide practical	Inner setting:	-	Educate: Conduct educational

	training for clinicians	Readiness for implementation -Access to knowledge and information		meetings<br><br>*The educational meeting will cover all practical aspects of the PROM process (e.g., administering, interpreting) as well as explain the justification for their use and expected benefits.*<br><br>Educate: Conduct ongoing training<br><br>Educate: Provide ongoing consultation
	Invest sufficient available resources in supporting the PROM process	Inner setting: Readiness for implementation -Available resources	An award-winning smartphone app and patient portal was adapted to be used to administer the PROM, improving the accessibility and management of PROM data.<br><br>To identify ways of addressing issues raised by the PROM, a prior study was conducted with patient and provider involvement (Intervention Pathways Study).	Educate: Conduct educational meetings<br><br>*Managing issues raised by the PROM will be addressed in educational meetings, drawing on the results of the Intervention Pathways Study and knowledge of local services.*<br><br>Educate: Provide ongoing consultation<br><br>*The Application Manager will provide ongoing support, including PROM data quality and systems monitoring.*
**Commencing**: Organization starting to use it	Provide clinicians opportunities to test out using the PROM and become confident using it before it is fully rolled out	Intervention characteristics: Trialability<br><br>Characteristics of individuals: Self-efficacy	As a pilot study to a more definitive trial, it inherently provides a period of trialability. It is a small-scale test of the intervention that will allow for refinement of the processes involved.	Quality management: Conduct cyclical small tests of change<br><br>*See all strategies aimed to educate.*
	Be prepared for issues to arise when starting to use the PROM	Process: Executing	-	Quality management: Conduct cyclical small tests of change<br><br>*Building on feedback received from patients and physicians and the Application Manager's field notes, we will use a rapid-cycle evaluation approach, using the CFIR, to improve implementation.*<br><br>Educate: Provide ongoing consultation
**Reflecting and developing**: Reflecting on the process	Organizations must spend time reflecting and evaluating the implementation	Process: Reflecting & evaluating	The pilot trial was designed to use mixed methods (quantitative and qualitative) to evaluate the progress and quality of the	Quality management: Conduct cyclical small tests of change<br><br>*Building on feedback received from patients and physicians and*

and making improvements	process.		PROM intervention's implementation.	*the Application Manager's field notes, we will use a rapid-cycle evaluation approach, using the CFIR, to improve implementation.*
	Ensure open channels of communication with time and space for clinicians to criticize and feedback on the PROM process	Process: Reflecting & evaluating	-	Plan: Organize clinician implementation team meetings
	Implementation leads must consider feedback and use it to develop the process	Process: Reflecting & evaluating	-	Quality management: Conduct cyclical small tests of change<br><br>*Building on feedback received from patients and physicians and the Application Manager's field notes, we will use a rapid-cycle evaluation approach, using the CFIR, to improve implementation.*

Note. PROM = patient-reported outcome measure; CFIR = Consolidated Framework for Implementation Research; * Reproduced or adapted from Foster et al. (2018); ** Based on Damschroder et al. (2009); *** Based on the taxonomies of Powell et al. (2012, 2015).

Table 3. Implementation science metrics and effectiveness outcomes collected for the pilot study.

**Objective**	**Construct**	**Data collected**	**Threshold for success**	**Participant group**		**Timing**
				Patients	Physi-cians	
Objective 1 -Evaluate perceptions of the I-Score innovation	Acceptability	***Primary outcome***:<br>Acceptability E-Scale (Tariman et al., 2011)	Score<br>$M \geq 24$	✓	✓	T1, T2, T3
		% likely to recommend the I-Score (NHS, 2014)	≥ 80%	✓	✓	T1, T2, T3
		Net Promoter Score (NHS, 2014)	> 0	✓	✓	T1, T2, T3
	Appropriate-ness	Perceived compatibility subscale (Moore and Benbasat, 1991)	Score<br>$M \geq 5.5$	-	✓	T1, T2, T3
		Appropriateness of Intervention Measure (Weiner et al., 2017)	Score<br>$M \geq 4$	✓	✓	T1, T2, T3
	Feasibility	Consent rate (and reasons for refusal)	≥ 70%	✓	✓	T1
		Retention rate	≥ 80%	✓	✓	T1, T2, T3
		Missing PROM (I-Score) data rate (e.g., due to non-completion, network failure)	≥ 90%	✓	✓	T1-T3
		Feasibility of Intervention Measure (Weiner et al., 2017)	Score<br>$M \geq 4$	✓	✓	T1, T2, T3
	Fidelity	% patients who complete the I-Score on time	≥ 90%	✓	-	T1, T2, T3
		% physicians who review the I-Score results on time	≥ 90%	-	✓	T1, T2, T3
Objective 2 - Evaluate the implement-tation strategy	Acceptability	Barriers and facilitators to implementation, based on the qualitative data collected*	-	✓	✓	T1-T3
	Appropriate-ness	Perceived fit of the implementation strategy within the clinic, based on the qualitative data collected*	-	✓	✓	T1-T3
	Feasibility	% of included physicians participating in the implementation activities (educational meeting, focus groups)	≥ 80%	-	✓	T1, T2, T3
		Rate of technical issues, based on the Application Manager's notes	-	-	-	T1-T3
	Fidelity	How and why the implementation strategy was adapted, based on the qualitative data collected*	-	✓	✓	T1-T3
Objective 3 – Determine preliminary intervention effective-ness & outcome measure utility	Patient management	Checklist of physician actions following review of the I-Score results	$p \leq 0.05$	-	✓	T1, T2, T3
	Barriers to ART adherence	The I-Score PROM	$p \leq 0.05$	✓	-	T1, T2, T3
	Adherence to ART	Self-Rating Scale Item (Feldman et al., 2013)	$p \leq 0.05$	✓	-	T1, T2, T3
	Viral load	The HIV RNA viral load, as indicated in the patient's medical file (> 50 copies = detectable)	$p \leq 0.05$	✓	-	T1, T3

PROM: patient-reported outcome measure; ART: antiretroviral therapy. * Qualitative data include the Application Manager's notes (T1-T3), the qualitative interviews with patients and the focus groups with physicians (T2, T3).

Appendix

Physician email invitation

Hello Dr. __________,

On behalf of Dr. Lebouché, we are inviting you to participate in the CTNPT039 study conducted at the CVIS.

The objective of this study is to pilot the award-winning Opal smartphone application to administer a new patient-reported outcome measure (ePROM) developed by our team for use in routine HIV care.

This ePROM captures patient-identified barriers to antiretroviral therapy adherence, the scores of which will be shared with their treating physician.

Your participation would involve having 6 of your patients complete the ePROM prior to three visits with you, spaced by 3-months and providing feedback on your experience.

We hope you will seriously consider being a part of this study.

Please contact me or Dr. Lebouché, to learn more.

[Coordinator name, telephone number]

Refusal form

1- What is your year of birth? __________

2- What is your sex?
- ☐ Female
- ☐ Male
- ☐ Trans

3- What is your preferred language?
- ☐ French
- ☐ English
- ☐ Other (please specify): ____________________

4- What is the main reason why you do not wish to participate in this study?

- ☐ I am not comfortable using smartphone apps
- ☐ I am concerned about confidentiality
- ☐ I am not interested in this study
- ☐ I do not have the time
- ☐ Other (please specify): ____________________

Time 1 questionnaire for people living with HIV

Your sociodemographic characteristics

The following questions concern your sociodemographic characteristics.

5- What is your year of birth? __________

6- What is your sex?
- ☐ Female
- ☐ Male
- ☐ Trans

7- What is your preferred language?
- ☐ French
- ☐ English
- ☐ Other (please specify): ____________________

8- What is the highest level of education that you have completed?
- ☐ Primary
- ☐ Secondary (High school)/Professional degree
- ☐ College/ Cegep/ Technical degree
- ☐ University
- ☐ Other (please specify): ____________________

9- What is your annual income ($CAD)?
- ☐ Less than $10,000
- ☐ $10,000-$19,999
- ☐ $20,000-$39,999
- ☐ $40,000-$59,999
- ☐ $60,000-$79,999
- ☐ $80,000-$99,999
- ☐ Greater than $100,000

10- Did you immigrate to this country?
- ☐ Yes
- ☐ No

11- What ethnic group(s) or family background(s) do you identify with? Select all that apply.
- ☐ Aboriginal or Indigenous
- ☐ English Canadian
- ☐ French Canadian
- ☐ French
- ☐ British
- ☐ Other Easter/Western European
- ☐ East Asian
- ☐ South Asian
- ☐ West Asian
- ☐ Arab or North African
- ☐ Latin American
- ☐ African
- ☐ Black
- ☐ Caribbean
- ☐ Pacific

- ☐ Mixed race/ethnicity
- ☐ Other (please specify): ____________________

12- Which of the following best describes your sexual orientation?
- ☐ Heterosexual
- ☐ Homosexual
- ☐ Bisexual

Your HIV diagnosis and medication

The next two questions concern your HIV diagnosis and HIV medication.

13- What year were you diagnosed with HIV? __________

14- How would you rate your overall satisfaction with your current HIV medication?

- ☐ 1—very dissatisfied
- ☐ 2
- ☐ 3
- ☐ 4
- ☐ 5—very satisfied

Your use of mobile devices

These next questions concern your use of mobile devices.

15- With what frequency do you use mobile devices (smartphone, tablet computer, cell phone, smartwatch, pocket PC, etc.)?
- ☐ Several times a day
- ☐ Once a day
- ☐ Several times per week
- ☐ Several times per month

16- On your tablet or smartphone, do you have any ‘apps’ related to health and wellness?
- ☐ Yes
- ☐ No

17- To what extent do you use applications related to health on a mobile device?
- ☐ Several times a day
- ☐ Once a day
- ☐ Several times per week
- ☐ Several times per month
- ☐ Once a month or less
- ☐ Never

Please indicate your level of agreement with the following statements.

18- I am confident that I can effectively report medical information using mobile technology.
- ☐ Strongly agree
- ☐ Agree
- ☐ Neutral
- ☐ Disagree
- ☐ Strongly disagree

19- If any health care provider asks me to report personal health data using a mobile device app, I will do so.

- ☐ Strongly agree
- ☐ Agree
- ☐ Neutral
- ☐ Disagree
- ☐ Strongly disagree

Your thoughts on using the I-Score

In this section, the questions are about your thoughts on using the I-Score measure, as completed through the Opal app.

2. How easy was the I-Score for you to use?
- ☐ 1—very difficult
- ☐ 2
- ☐ 3
- ☐ 4
- ☐ 5—very easy

3. How understandable were the questions?
- ☐ 1—difficult to understand
- ☐ 2
- ☐ 3
- ☐ 4
- ☐ 5—easy to understand

4. How much did you enjoy using the I-Score?
- ☐ 1—not at all
- ☐ 2
- ☐ 3
- ☐ 4
- ☐ 5—very much

5. How helpful was the I-Score in describing difficulties experienced taking HIV medication?
- ☐ 1—very unhelpful
- ☐ 2
- ☐ 3
- ☐ 4
- ☐ 5—very helpful
- ☐ Not applicable

6. Was the amount of time it took to use the I-Score acceptable?
- ☐ 1—very unacceptable
- ☐ 2
- ☐ 3
- ☐ 4
- ☐ 5—very acceptable

7. How would you rate your overall satisfaction with the I-Score?
- ☐ 1—very dissatisfied
- ☐ 2
- ☐ 3
- ☐ 4
- ☐ 5—very satisfied

8. How likely are you to recommend the I-Score?
- ☐ Extremely unlikely
- ☐ Unlikely
- ☐ Neither likely nor unlikely
- ☐ Likely
- ☐ Extremely likely

9. Using the I-Score seems…	Completely disagree	Disagree	Neither agree nor disagree	Agree	Completely agree
a. fitting	①	②	③	④	⑤
b. suitable	①	②	③	④	⑤
c. applicable	①	②	③	④	⑤
d. like a good match	①	②	③	④	⑤
e. implementable	①	②	③	④	⑤
f. possible	①	②	③	④	⑤
g. doable	①	②	③	④	⑤
h. easy	①	②	③	④	⑤

Adherence to your HIV medication

10. Rate your ability to take all your HIV medications as prescribed, over the past 4 weeks.
- ☐ Very poor
- ☐ Poor
- ☐ Fair
- ☐ Good
- ☐ Very good
- ☐ Excellent

Comments?

____

____

____

Thank you!

Time 1 questionnaire for HIV physicians

Your sociodemographic characteristics and HIV practice

The following five questions concern your sociodemographic characteristics and HIV practice.

1- What is your year of birth? ___________

2- What is your sex?
- ☐ Female
- ☐ Male

3- What is your preferred language?
- ☐ French
- ☐ English
- ☐ Other (please specify): ______________________

4- How long have you been treating people with HIV?
- ☐ 1-4 years
- ☐ 5-9 years
- ☐ 10-14 years
- ☐ 15-19 years
- ☐ 20 years or more

5- Approximately how many people with HIV are you currently following? ___________

Your thoughts on using the I-Score

These last questions are about your thoughts on using the I-Score measure, as accessed by you through the ORMS dashboard.

6- How easy was the I-Score for you to use?

- ☐ 1—very difficult
- ☐ 2
- ☐ 3
- ☐ 4
- ☐ 5—very easy

7- How understandable were the questions?

- ☐ 1—difficult to understand
- ☐ 2
- ☐ 3
- ☐ 4
- ☐ 5—easy to understand

8- How much did you enjoy using the I-Score?
- ☐ 1—not at all
- ☐ 2
- ☐ 3
- ☐ 4
- ☐ 5—very much

9- How helpful was the I-Score in describing difficulties experienced taking HIV medication?

- ☐ 1—very unhelpful
- ☐ 2
- ☐ 3
- ☐ 4
- ☐ 5—very helpful
- ☐ Not applicable

10- Was the amount of time it took to use the I-Score acceptable?

- ☐ 1—very unacceptable
- ☐ 2
- ☐ 3
- ☐ 4
- ☐ 5—very acceptable

11- How would you rate your overall satisfaction with the I-Score?

- ☐ 1—very dissatisfied
- ☐ 2
- ☐ 3
- ☐ 4
- ☐ 5—very satisfied

12- How likely are you to recommend the I-Score?

- ☐ Extremely unlikely
- ☐ Unlikely
- ☐ Neither likely nor unlikely
- ☐ Likely
- ☐ Extremely likely

13- Using the I-Score seems…	Completely disagree	Disagree	Neither agree nor disagree	Agree	Completely agree
a. fitting	①	②	③	④	⑤
b. suitable	①	②	③	④	⑤
c. applicable	①	②	③	④	⑤
d. like a good match	①	②	③	④	⑤
e. implementable	①	②	③	④	⑤
f. possible	①	②	③	④	⑤
g. doable	①	②	③	④	⑤
h. easy	①	②	③	④	⑤

14- Using the I-Score is compatible with all aspects of my work

- ☐ Extremely disagree
- ☐ Disagree
- ☐ Somewhat disagree
- ☐ Neither agree nor disagree
- ☐ Somewhat agree
- ☐ Agree
- ☐ Extremely agree

15- Using the I-Score is completely compatible with my current work situation

- ☐ Extremely disagree
- ☐ Disagree
- ☐ Somewhat disagree
- ☐ Neither agree nor disagree
- ☐ Somewhat agree
- ☐ Agree
- ☐ Extremely agree

16- I think that using the I-Score fits well with the way I like to work

- ☐ Extremely disagree
- ☐ Disagree
- ☐ Somewhat disagree
- ☐ Neither agree nor disagree
- ☐ Somewhat agree
- ☐ Agree
- ☐ Extremely agree

17- Using the I-Score fits into my work style

- ☐ Extremely disagree
- ☐ Disagree
- ☐ Somewhat disagree
- ☐ Neither agree nor disagree
- ☐ Somewhat agree
- ☐ Agree
- ☐ Extremely agree

Comments?

____

____

____

Thank you!

Time 2 and Time 3 questionnaire for people living with HIV

Your thoughts on using the I-Score

In this section, the questions are about your thoughts on using the I-Score measure, as completed through the Opal app. Please answer these questions thinking about your most recent use of the I-Score.

1- How easy was the I-Score for you to use?

- ☐ 1—very difficult
- ☐ 2
- ☐ 3
- ☐ 4
- ☐ 5—very easy

2- How understandable were the questions?

a. 1—difficult to understand
b. 2
c. 3
d. 4
e. 5—easy to understand

3- How much did you enjoy using the I-Score?

a. 1—not at all
b. 2
c. 3
d. 4
e. 5—very much

4- How helpful was the I-Score in describing difficulties experienced taking HIV medication?

a. 1—very unhelpful
b. 2
c. 3
d. 4
e. 5—very helpful
f. Not applicable

5- Was the amount of time it took to use the I-Score acceptable?

a. 1—very unacceptable
b. 2
c. 3
d. 4
e. 5—very acceptable

6- How would you rate your overall satisfaction with the I-Score?

a. 1—very dissatisfied
b. 2
c. 3
d. 4
e. 5—very satisfied

7- How likely are you to recommend the I-Score?
- ☐ Extremely unlikely
- ☐ Unlikely
- ☐ Neither likely nor unlikely
- ☐ Likely
- ☐ Extremely likely

8- Using the I-Score seems…	Completely disagree	Disagree	Neither agree nor disagree	Agree	Completely agree
a. fitting	①	②	③	④	⑤
b. suitable	①	②	③	④	⑤
c. applicable	①	②	③	④	⑤
d. like a good match	①	②	③	④	⑤
e. implementable	①	②	③	④	⑤
f. possible	①	②	③	④	⑤
g. doable	①	②	③	④	⑤
h. easy	①	②	③	④	⑤

Adherence to your HIV medication

9- Rate your ability to take all your HIV medications as prescribed, over the past 4 weeks.
- ☐ Very poor
- ☐ Poor
- ☐ Fair
- ☐ Good
- ☐ Very good
- ☐ Excellent

Comments?

____

____

____

Thank you!

Time 2 and Time 3 questionnaire for HIV physicians

Your thoughts on using the I-Score

In this questionnaire, we ask about your thoughts on using the I-Score measure, as accessed by you through the ORMS dashboard. Please answer these questions thinking about your most recent uses of the I-Score.

10- How easy was the I-Score for you to use?
  a. 1—very difficult
  b. 2
  c. 3
  d. 4
  e. 5—very easy

11- How understandable were the questions?
  a. 1—difficult to understand
  b. 2
  c. 3
  d. 4
  e. 5—easy to understand

12- How much did you enjoy using the I-Score?
  a. 1—not at all
  b. 2
  c. 3
  d. 4
  e. 5—very much

13- How helpful was the I-Score in describing difficulties experienced taking HIV medication?
  a. 1—very unhelpful
  b. 2
  c. 3
  d. 4
  e. 5—very helpful
  f. Not applicable

14- Was the amount of time it took to use the I-Score acceptable?
  a. 1—very unacceptable
  b. 2
  c. 3
  d. 4
  e. 5—very acceptable

15- How would you rate your overall satisfaction with the I-Score?
  a. 1—very dissatisfied
  b. 2
  c. 3
  d. 4
  e. 5—very satisfied

16- How likely are you to recommend the I-Score?
- ☐ Extremely unlikely
- ☐ Unlikely
- ☐ Neither likely nor unlikely
- ☐ Likely
- ☐ Extremely likely

17- Using the I-Score seems…	Completely disagree	Disagree	Neither agree nor disagree	Agree	Completely agree
a. fitting	①	②	③	④	⑤
b. suitable	①	②	③	④	⑤
c. applicable	①	②	③	④	⑤
d. like a good match	①	②	③	④	⑤
e. implementable	①	②	③	④	⑤
f. possible	①	②	③	④	⑤
g. doable	①	②	③	④	⑤
h. easy	①	②	③	④	⑤

18- Using the I-Score is compatible with all aspects of my work
- ☐ Extremely disagree
- ☐ Disagree
- ☐ Somewhat disagree
- ☐ Neither agree nor disagree
- ☐ Somewhat agree
- ☐ Agree
- ☐ Extremely agree

19- Using the I-Score is completely compatible with my current work situation
- ☐ Extremely disagree
- ☐ Disagree
- ☐ Somewhat disagree
- ☐ Neither agree nor disagree
- ☐ Somewhat agree
- ☐ Agree
- ☐ Extremely agree

20- I think that using the I-Score fits well with the way I like to work

- ☐ Extremely disagree
- ☐ Disagree
- ☐ Somewhat disagree
- ☐ Neither agree nor disagree
- ☐ Somewhat agree
- ☐ Agree
- ☐ Extremely agree

21- Using the I-Score fits into my work style

- ☐ Extremely disagree
- ☐ Disagree
- ☐ Somewhat disagree
- ☐ Neither agree nor disagree
- ☐ Somewhat agree
- ☐ Agree
- ☐ Extremely agree

Comments?

____

____

____

Thank you!

Physician checklist per HIV patient visit

Date:

Day Month Year

Patient ID:

Visit: ☐ Time 1 ☐ Time 2 ☐ Time 3

**At this visit, did you….**	**Yes**	**No**
Receive the I-Score results on time (prior to or during the visit)?	☐	☐
Review the I-Score results prior to or during the visit?	☐	☐
Discuss the I-Score results with your patient?	☐	☐
Identify an adherence barrier of concern with the I-Score results?	☐	☐

Based on the I-Score results, did you do any of the following?
(Check all that apply)

Record issues in your medical notes	☐
Provide advice, information, or education	☐
Refer the patient to another health or social service provider	☐
Order a test	☐
Modify a medication or treatment	☐
Other:	☐
**No/no action was required**	☐

Comments

Qualitative interview schedule 1 (Time 2)

The purpose of this interview is to learn about your experience using the I-Score, a measure of patient-reported barriers to HIV medication adherence. This includes your experience with the Opal patient portal.

The information you provide will help us to understand how we can facilitate the use of the I-Score and Opal.

1- What was it like for you to learn to use the I-Score and Opal?
- What made it difficult to learn to use them?
- What made it easy to learn to use them?

2- What do you think about the support or training you received to use them?
- How could it be improved?

3- In general, what problems did you face?
- What could have helped?

4- How do you feel about the quality of the information provided by the I-Score?
- What was most helpful about the I-Score?
- What could make the I-Score more helpful?

5- What impact, if any, did the I-Score and Opal have on the care provided?
- …on your work (physicians)?

6- What would you change about the I-Score or Opal?
- What would make using them easier for you?

7- Overall, how satisfied are you with the I-Score and Opal?

Qualitative interview schedule 2 (Time 3)

The purpose of this interview is to learn more about your experience using the I-Score, a measure of patient-reported barriers to HIV medication adherence. This includes your experience with the Opal patient portal.

The information you provide will help us to understand how we can facilitate the use of the I-Score and Opal.

Since our last focus group (or interview) ...

1- What might have changed in the way you use the I-Score and Opal?
- To what extent do you use the I-Score and Opal differently than when you first started using them?

2- What problems have you faced?
- How were they dealt with?
- What could have helped?

3- What do you think about the support you had to use the I-Score and Opal?
- How could it be improved?

4- How do you feel about the quality of the information provided by the I-Score?

5- What impact, if any, did the I-Score and Opal have on the care provided?
- …on your work (physicians)?

6- What would you change about the I-Score or Opal?
- What would make using them easier for you?

7- Overall, how satisfied are you with the I-Score and Opal?

INVESTIGATOR AGREEMENT

Protocol Title: The I-Score/Opal Implementation Pilot Study

Implementation of an electronic patient-reported measure of barriers to antiretroviral therapy adherence with the Opal patient portal: a mixed method type 3 hybrid pilot study at a large Montreal HIV clinic

Date of protocol: 16 december 2020

This clinical study will be conducted in accordance with applicable Health Canada regulations, ICH guidelines on current GCP, and the Declaration of Helsinki.

I confirm that I have read and understand this protocol and all appendices, and I agree to conduct this clinical study in accordance with the design and specific provisions of the protocol, with the exception of a change intended to eliminate an immediate hazard to participants. Any deviation from the study protocol will be documented in the case report form.

I agree to promptly report to the applicable ethics boards any changes in the research activity and all unanticipated problems involving risks to human participants or others. Additionally, I will not make any changes in the research without prior ethics and sponsor approval, except where necessary to ensure the safety of study participants.

Name	Signature	Date (dd-mmm-yyyy)
