# Supplementary material for: Implementation of an electronic patient-reported measure of barriers to antiretroviral therapy adherence with the Opal patient portal: Protocol for a mixed method type 3 hybrid pilot study at a large Montreal HIV clinic
Source: PLoS One. 2021 Dec 30;16(12):e0261006. doi: 10.1371/journal.pone.0261006 (PMC8717992; doi:10.1371/journal.pone.0261006)
Supplement: Attachment — Submitted filename: Response to Reviewers.pdf [file pone.0261006.s007.pdf]

June 8, 2021

Ethan Moitra
Academic Editor
PLOS ONE
Brown University

Reviewers 1 & 2

Response to Reviewers

Dear Dr Moitra and Reviewers,

On behalf of the authors, I thank you for the opportunity to address the reviewers' thoughtful comments, suggestions, and questions. A response to each, in turn, appears below. We also respond to the journal requirements that needed greater consideration.

We hope that the changes brought to the manuscript are satisfactory and look forward to receiving your final decision.

Sincerely,

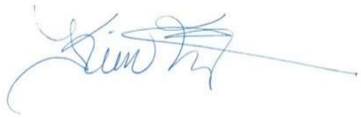

Kim Engler
Center for Outcomes Research & Evaluation
Research Institute, McGill University Health Centre
Montreal, Quebec, Canada

**Comment**	**Author response**
Academic editor	
Please review your **reference list** to ensure that it is complete and correct. Any changes to the reference list should be mentioned in the rebuttal letter that accompanies your revised manuscript.	The reference list was checked in relation to the text and one correction was made.<br><br>Three new references were added to address Reviewer #2's comments. They are numbered 66 to 68 and are as follows:<br>- Williams AB, Amico KR, Bova C, Womack JA. A proposal for quality standards for measuring medication adherence in research. AIDS Behav. 2013;17(1):284-297.<br>- Wilson IB, Carter AE, Berg KM. Improving the self-report of HIV antiretroviral medication

Comment	Author response
	adherence: is the glass half full or half empty? Curr HIV/AIDS Rep. 2009 Nov;6(4):177-86.<br>- Greenhalgh J, Gooding K, Gibbons E, Dalkin S, Wright J, Valderas J, *et al.* How do patient reported outcome measures (PROMs) support clinician-patient communication and patient care? A realist synthesis. J Patient Rep Outcomes. 2018;2:42.
Please ensure that your manuscript meets **PLOS ONE's style requirements**, including those for file naming.	We reexamined the 'Manuscript body formatting guidelines' and made some adjustments to the text and tables.
Please provide an amended **Competing Interests Statement** that explicitly states this commercial funder [Merck Canada Inc.], along with any other relevant declarations relating to employment, consultancy, patents, products in development, marketed products, etc.<br><br>Within this Competing Interests Statement, please confirm "This does not alter our adherence to PLOS ONE policies on sharing data and materials."<br><br>Please include your amended Competing Interests Statement within your cover letter. We will change the online submission form on your behalf.	I could not find the original "Competing Interests Statement", but one is provided below with the requested amendments and more details on funding sources.<br><br>This study secured funding for its conduct from the Canadian Institutes of Health Research HIV Clinical Trials Network (grant # CTNPT 039, 2019-2021), Merck Canada Inc./ MSD France (Investigator-Initiated Study Program (IISP) grant # IISP-53538, 2015-2021) as well as MSD Avenir/ Assistance Publique–Hôpitaux de Paris (AP-HP) Foundation (grant # DS-2018-0072). BL has received research support and consulting fees from ViiV Healthcare, Merck, and Gilead. This does not alter our adherence to PLOS ONE policies on sharing data and materials.
We note that you have stated that you will provide repository information for your data at acceptance. Should your manuscript be accepted for publication, we will hold it until you provide the relevant accession numbers or DOIs necessary to access your data. If you wish to make changes to your **Data Availability statement**, please describe these changes in your cover letter and we will update your	As this is a protocol, it is unclear how we should manage the issue of data access since no data is yet available and no findings will be presented in the manuscript. Data access seems more relevant upon publishing the pilot study results which we can surely grant; however, at this time, we cannot confirm where this information will be stored. Perhaps we could state instead that:<br><br>The quantitative data will be made available upon publication of the study results and details as to their location will be provided at that time.

Comment	Author response
Data Availability statement to reflect the information you provide.<br><br>The PLOS Data policy requires authors to make all data underlying the findings described in their manuscript fully available without restriction, with rare exception, at the time of publication.	

Reviewer #1

Comment	Author response
It may be good to use the term protocol in your title since it isn't immediately clear.	Agreed. The titles now read:<br><br>Implementation of an electronic patient-reported measure of barriers to antiretroviral therapy adherence with the Opal patient portal: protocol for a mixed method type 3 hybrid pilot study at a large Montreal HIV clinic<br><br>Short title:<br>Electronic capture of adherence barriers for HIV care: a pilot study protocol
It would be good to keep consistent with the use of acronyms. Sometimes you use PROM and sometimes you use "patient-reported outcome measures" (e.g. on line 87 and on line 97)	Agreed. The manuscript was revised to consistently use the acronym PROM.
Line 134 - You mention how many patients are in the HIV clinic, but it would also be good to mention how many physicians are in the clinic. It is interesting to know since you mention your sample size for the pilot will be 5 physicians.	We have added this information on line 146: "Among the 16 physicians actively treating individuals with HIV at the CVIS,…"
Line 139 - Is there a inclusion criteria related to length of time on ART? You mention later that participants have to have signs of adherence problems within the last 12 months, but it wasn't clear if they had to have been taking ART for that long.	No, there is not. We now clarify this on line 149: "…on combination ART, irrespective of duration,…"
Line 156 - Many studies have physicians involved in recruiting	Yes, that is a concern. It is why it is the study coordinator who will present the study in detail and

Comment	Author response
their patients, but I just wondered if you had considered the possible ethical issues associated with this? Patients may feel undue pressure or obligation to participate.	manage the consent process. The physician's involvement is limited to: 1) selecting patients of theirs that a neutral staff member can contact to ascertain interest in the study; or 2) asking a patient if they would like to know more about the study. Subsequently, it is the study coordinator who would present the study more fully to potential participants.<br><br>The relevant section was revised to make this clear. It now reads as follows, beginning on line 163:<br><br>"Patients of participating physicians will be recruited and consented in two ways: 1) when they visit the clinic; or 2) prior to an upcoming clinic visit. When they visit the clinic to meet with a health or social service provider (e.g., physician, nurse, social worker, psychologist), the provider will briefly describe the study to determine interest. Alternatively, suitable patients with upcoming visits will be identified by the physician and then contacted by a neutral clinic staff member to inform them of the study. If the individual is interested, the study coordinator will present the project in greater detail to them in person or over the phone, check the eligibility criteria, and obtain consent."
Line 193 - Will the Application Manager be the one meeting with the patients/providers before T1 to train them?	Exactly. So that this is very clear, we have adjusted the sentence beginning on Line 207: "The AM will train patients and providers and be available to them on an ongoing basis, as needed, preferably by phone or teleconference."
Line 207 - With the cyclical tests of change, does this mean the process may be adapted for participants e.g. from T2 to T3? Would the change occur for all participants or be on an individual basis?	Essentially, adaptations could occur between T1 and T3. Adaptations will be discussed with the Team on a case-by-case basis and depend on the limits of the technology and what is manageable within the setting. For example, maybe it would be possible, if the need presents itself, to individualize the reminders (e.g., for when to complete the PROMs) rather than having them occur at a fixed time for all.
More detail on the researchers involved would be useful and interesting. Who will be running the focus groups? Who and how many researchers will be analysing the data? How will disagreements between researchers be handled? Particularly for qualitative data,	Unfortunately, at this time, we cannot specify who on our extended team will be involved in the qualitative analyses. Several individuals are trained in qualitative data collection and analysis and would be qualified for the tasks. This will depend on their availability. We have, however, sought to answer your questions more generally below. When publishing the results of this study, we will provide more background on those

Comment	Author response
having an overview of the background of the researchers is important.	involved in the qualitative analysis and details on its process.<br><br>Line 250: "Focus groups and patient interviews will be conducted by an experienced interviewer…"<br>Line 397: "Two trained coders will be involved in the analyses which will be discussed during periodic team meetings, including any discrepancies in coding or interpretation."
Table 1 (Line 238) - At the very end it mentions compensation for the patients, but I was unable to find any detail on what this compensation was.	The specific compensation is provided in the patient consent form in the Appendix. It is described as follows (funds are in Canadian dollars):<br>"You will receive $20 each time you complete the online research questionnaire, that is: at baseline, at 3 months and at 6 months into the study, for a total of $60. If you are invited to participate in a one-on-one interview, you will be compensated $30 for each interview you participate in. If you choose to stop taking part in the study or are removed from it before the study is completed, you will be paid only part of this money depending on the length of time you took part."
When reviewing Table 2 (Line 248), I was wondering how long approximately will the questionnaires take at the different times points for both patients and physicians?	We have yet to pilot the questionnaires so a precise estimate of the time it will take to complete them is not available. This is one reason why we included the questionnaires in appendix 2. However, based on previous research with Opal (for use in COVID-19 remote follow-up), it took patient participants on average 10 to 15 seconds per question, when filling out a questionnaire for the first time. Depending on the time period, people with HIV will have from 21 to 31 questions to answer, while physicians will have from 19 to 24. Hence, it should take at the most about 8 minutes to complete each questionnaire. This information has been added to the manuscript, beginning on Line 234:<br><br>"At T1, the study questionnaire will be composed of 31 questions for patients and 24 for physicians, while at T2 and T3, it will have 21 questions for patients and 19 for physicians. Based on the metadata of patients who have used Opal, these questionnaires should take less than 10 minutes to complete, considering that patients take 10-15 seconds per question, at first completion."<br><br>The I-Score measure will always be completed by patients prior to meeting their physician. As to when the

Comment	Author response
It was also not exactly clear whether the questionnaires were filled in before the patient meets their physician or afterwards at each time point.	study questionnaires are to be completed, this information is now provided in parentheses, in Table 1:<br>- Complete the online study questionnaire (after the clinic visit (s))<br>- Possibly participate in an online qualitative interview (after the clinic visit)<br>- Participate in an online focus group (after several clinic visits with participating patients)
Line 330 - change qualitative to quantitative	Qualitative is actually correct. To evaluate the implementation strategy (and not perceptions of the intervention), the assessment of acceptability, appropriateness, and fidelity will be based on qualitative data (see Table 2).
You do mention that you have published on the barriers measure elsewhere, but it would have been good to have included a little more detail on this - how many barriers and the type of barriers it covers.	More information on the content domains of the I-Score is now provided, beginning on Line 184:<br><br>"The I-Score PROM contains 20 items, covering 6 domains of barriers to ART adherence: cognitive and emotional aspects; lifestyle factors; the social and material context; the health experience and state; characteristics of ART; and the healthcare system and its services.<br><br>I have also included example items. See Line 188:<br><br>"Example items include "I was not motivated to take my medication," "I felt isolated or alone," "I had another health condition to deal with (for example, depression, diabetes, or heart disease)," and "My medication cost coverage was not sufficient.""

Reviewer #2

It is a awkward to write (ln. 49) that PROMs are rarely used in clinical practice and then state (ln. 54) that evidence for PROM is mixed and then beneficial in the following paragraph. Unclear if referring to PROMs overall or PROMs related to HIV.	Agreed. We have tried to reduce the ambiguity, with some changes. In the upcoming passage, we are referring to research on the use of PROMs in care, across care specialties. This does not conflict with PROMs rarely being used in HIV clinical practice. Hopefully, this resolves the awkwardness.<br><br>Line 60: "While <u>past</u> syntheses of effectiveness evidence for PROM use <u>across specialties</u> in routine care have typically found mixed results, with inconsistent impacts on patient outcomes […] a more recent systematic review published in 2019 finds the evidence supports PROM use in standard care…"

Comment	Author response
The team is investigating barriers to ART adherence and cite some recently literature on the measurement of this topic. However, as others have shown, one major limitation is relying on participants to describe what are their most important barriers, which may be based on convenience and social desirability. For example, studies show that the #1 barrier to ART adherence is "simply forgetting," irrespective of viral load, thus, it could be that simply forgetting is a consequence of neurocognitive functioning, depression, or social desirability in not wanting to report substance use. The promise of **a PROM intervention must take into account why PLWH report the barriers they do** as you may miss the targets that truly drive non-adherence.	Thank you for raising these very important points. Missing the target is indeed a risk with the proposed intervention. We have tried to take account of this in several ways: 1) We understand barriers to be multidimensional, complex, and not necessarily mutually exclusive. Our previous work on this, exemplifies this well (Engler et al. AIDS Care. 2018;30(Sup1):17-28).; 2) Therefore, we do not expect the PROM to provide the full portrait of a patient's adherence barriers (especially not one with 20 items, when we identified 100 initially, each corresponding to a different barrier). One of its purposes is to help instigate and guide conversations between patients and providers. Hence, we assume that a discussion around forgetting may eventually lead to the source of this forgetting (whether due to stigma management, like hiding meds; unexpected schedule changes; the other aspects mentioned, etc.); 3) We are administering the PROM electronically which may foster more honest reporting; 4) We have sought to involve patients throughout the development of this PROM. For instance, we have an HIV patient committee including a past substance user, which has been active since 2016, which has given their input on the conceptual framework of the instrument. We conducted a Delphi study with 40 people living with HIV and 57 HIV care providers who rated each of the PROM's possible items for relevance, retaining only those that both groups endorsed. We conducted 12 cognitive interviews with people living with HIV asking, for example, about the appropriateness of the questionnaire items and making adjustments accordingly; 5) Finally, we are conducting a pilot study with stakeholder engagement to learn more about the workings of the intervention in practice and hopefully improve it in the process.<br><br>In response to this important comment, we have added a paragraph to the Anticipated problems section, starting on line 455, along with some additional references.
